# SWATH-MS for prospective identification of protein blood biomarkers of rtPA-associated intracranial hemorrhage in acute ischemic stroke: a pilot study

**DOI:** 10.1038/s41598-021-97710-9

**Published:** 2021-09-21

**Authors:** Bartosz Karaszewski, Anna Gójska-Grymajło, Paulina Czaplewska, Bartosz Jabłoński, Aleksandra E. Lewandowska, Daria Ossowska, Adam Wyszomirski, Marek Hałas, Edyta Szurowska

**Affiliations:** 1 Department of Adult Neurology, Medical University of Gdańsk & University Clinical Center, Gdańsk, Poland; 2 Intercollegiate Faculty of Biotechnology UG&MUG, University of Gdańsk, Gdańsk, PL Poland; 3 II Department of Radiology, Medical University of Gdańsk, Gdańsk, Poland

**Keywords:** Stroke, Proteomics

## Abstract

Intravenous recombinant tissue plasminogen activator (rtPA) is, besides mechanical thrombectomy, the highest class evidence based reperfusion treatment of acute ischemic stroke (AIS). The biggest concern of the therapy is symptomatic intracranial hemorrhage (sICH), which occurs in 3–7% of all treated patients, and is associated with worse functional outcome. Finding a method of the powerful identification of patients at highest risk of sICH, in order to increase the percentage of stroke patients safely treated with rtPA, is one of the most important challenges in stroke research. To address this problem, we designed a complex project to identify blood, neuroimaging, and clinical biomarkers combined for prospective assessment of the risk of rtPA-associated ICH. In this paper we present results of blood proteomic and peptide analysis of pilot 41 AIS patients before rtPA administration (the test ICH group, n = 9 or the controls, without ICH, n = 32). We demonstrated that pre-treatment blood profiles of 15 proteins differ depending on whether the patients develop rtPA-associated ICH or not. SWATH-MS quantification of serum or plasma proteins might allow for robust selection of blood biomarkers to increase the prospective assessment of rtPA-associated ICH over that based solely on clinical and neuroimaging characteristics.

## Introduction

Acute stroke, with ischemic stroke comprising 80% of all cerebrovascular incidents, has been recognized as one of the core problems in clinical medicine in need of prevention and treatment. It remains one of the most deleterious diseases that produce high social and economic costs worldwide. In 2016, of the 17.8 million deaths due to cardiovascular disease worldwide, 5.7 million were due to strokes^1^. In 2013, there were almost 25.7 million stroke survivors globally (71% with ischemic stroke) and 113 million disability adjusted life years due to stroke (58% due to ischemic stroke)^2^.

Introduction of acute reperfusion treatment: intravenous thrombolysis, and in the past 5 years—the mechanical thrombectomy, in combination with specific and general stroke unit procedures, have greatly improved functional outcome after ischemic stroke. Thrombolytic treatment with recombinant tissue plasminogen activator (rtPA) was the first breakthrough in the reperfusion, thus in some sense causative, stroke treatment. Intravenous rtPA is the mainstay and the highest class evidence based method of acute ischemic stroke treatment, and is currently recommended 0–4.5 h after stroke onset^3^. Moreover, some patients selected with dedicated neuroimaging and special clinical characteristics might be treated even beyond the standard time window^4^.

In most patients decision on i.v. rtPA administration is straightforward, however, in some cases it might be complex. The biggest concern is the symptomatic intracranial hemorrhage (sICH), which occurs in 3–7% of all treated patients, and is associated with worse 90-day functional outcome and higher disability than in those untreated^3,5^. There are various measures to quantify the extension of rtPA-associated ICH in clinical trials including the European Cooperative Acute Stroke Study (ECASS) scale (Table 1). Clinical measures used to quantify the neurological deterioration due to rtPA-related sICH include SITS-MOST (The Safe Implementation of Thrombolysis in Stroke-Monitoring Study) and ECASS III^3^. The former is defined as intracerebral hemorrhage classified as local or remote PH2 within 24–36 h after rtPA bolus administration with the clinically important deterioration of neurological status, whereas the latter is defined as any hemorrhage with clinical deterioration of 4 or more points on the National Institutes of Health Stroke Scale (NIHSS) score.

**Table 1:** The European Cooperative Acute Stroke Study scale of the hemorrhagic transformation after the thrombolytic treatment^43,44^.

Hemorrhage classification	Radiographic appearance
Hemorrhage infarction type 1 (HI1)	Small hyperdense petechiae
Hemorrhage infarction type 2 (HI2)	More confluent hyperdensity throughout the infarct zone; without mass effect
Parenchymal hematoma type 1 (PH1)	Homogeneous hyperdensity occupying < 30% of the infarct zone; some mass effect
Parenchymal hematoma type 2 (PH2)	Homogeneous hyperdensity occupying > 30% of the infarct zone; significant mass effect. Or, any homogenous hyperdensity located beyond the borders of the infarct zone

Meta-analysis of nine trials of intravenous rtPA administration versus control^6^ showed increased risk of PH2 [6.8% vs 1.3%, OR 5.55 (4.01–7.70)]. SITS-MOST registry also revealed higher frequency of PH2 [3.7% vs 0.6%, OR 6.67 (4.11–10.84)] and fatal intracerebral hemorrhage [2.7% vs 0.4%, OR 7.14 (3.98–12.79)].

Apart from the most common intraparenchymal hemorrhages anatomically related to ischemic lesion, in some patients treatment with i.v. rtPA is complicated by bleeding located remotely from the lesion, which may constitute a substantial portion of all ICHs and affect as many as 27.5% of patients^7^. However, there is little information on the prevalence of the remote-ICHs from large clinical trials on rtPA therapy for stroke.

Finding a method of the powerful (highly specific and selective) identification of patients at highest risk of sICH, in order to increase the percentage of stroke patients safely treated with rtPA, is one of the most important challenges in stroke research. The SITS-MOST identified nine independent risk factors for sICH: baseline NIHSS score, serum glucose, systolic blood pressure, history of hypertension, age, body weight, stroke onset to treatment time, aspirin monotherapy, and dual antiplatelet therapy with aspirin and clopidogrel. Unfortunately, any patterns or combinations of these and other characteristics into scoring systems (GRASPS, Glucose, Race, Age, Sex, Pressure, Stroke Severity; DRAGON (Dense Artery, Rankin Score, Age, Glucose, Onset to Treatment Time, National Institutes of Health Stroke Scale (NIHSS)), SEDAN (Sugar, Early Infarct Signs, Dense Artery, Age, NIHSS)) still perform poorly and have only modest predictive value for identifying patients at risk^8^.

To address this problem we designed a major and complex project to identify blood, neuroimaging, and clinical biomarkers combined for prospective assessment of the risk of intracranial hemorrhage (ICH) after thrombolytic treatment of acute ischemic stroke (Investigator Initiated Study funded from Siemens Healthineers, 2018, to B. Karaszewski, Medical University of Gdansk) with analysis of multiple-origin data with deep learning techniques (hypothesis-free approach). The study was designed to recruit 400 ischemic stroke patients treated with i.v. rtPA, but herein we present results of blood proteomic and peptide analysis of pilot 41 patients.

The proteomic approach has already been separately recognized as a valuable and comprehensive method enabling insights into the pathophysiology of stroke with proteomic profile assessed in the brain of stroke patients^9–12^, in the endothelial progenitor cells^13^, in platelets^14^ or in thrombi-emboli retrieved during the mechanical thrombectomy^15^. In 2015, a proteomics chip study on large Swedish PIVUS and ULSAM cohorts proved ten proteins to be related to the incident of stroke^16^. In another study on H-type hypertension related stroke, with use of iTRAQ-based LC–MS/MS proteomics approach, AT-3, CRP, ApoB, and AHSG were proved to be the strongest predictors of this type of stroke^17^. The same iTRAQ-based LC–MS approach was used in 50 stroke patients and 60 proteins showed a ≈1.5-fold change, with candidate proteins vWF, ADAMTS13, S100A7, and DLG4 confirmed through ELISA to corroborate with the experimental findings^18^. In the two phase SpecTRA study using liquid chromatography/multiple reaction monitoring-mass spectrometry, insulin-like growth factor-binding protein 3 and serum paraoxonase/lactonase 3 were found to be reliable and reproducible biomarkers for TIA in the Emergency Department settings^19^. In another study on TIA/minor stroke, ceruloplasmin, complement component C8 gamma (C8γ), and platelet basic protein were significantly different between the ischemic group (TIA and minor stroke) and the controls^20^ Finally, the SWATH method was used for analysis of serum of 20 ischemic stroke patients and 11 proteins were defined as candidate biomarkers^21^.

There are only two proteomic studies that concern rtPA treatment in ischemic stroke. In one of them plasma from acute stroke patients was analyzed pre- and post-intravenous tPA using tandem mass spectrometry and protein array profiling. The rtPA treated patients presented with distinct and elongated degradomic patterns in comparison to non-tPA treated patients^22^. In the second study, high-resolution mass spectrometry and long high-performance liquid chromatography were used to investigate changes in blood proteins after stroke and as a result of thrombolysis treatment. In this study ten patients were treated with rtPA and had up to 5 blood samples collected at different time points after stroke with 26 proteins being proved to be expressed differently and 23 proteins showing significant changes of expression over time^23^. However, up to date there have been no proteomic studies specifically confronting the possible biomarkers of hemorrhagic complications in stroke patients treated with rtPA, which make the release of the partial patient sample data reasonable. Due to its small size, and assumed biomolecular character of this paper, we do not here combine all individual data (clinical, neuroimaging, proteomic) into the scoring systems. Herein, we reveal our general methodological approach with shortlisting of blood peptide or protein candidates selected with Sequential Window Acquisition of All Theoretical Mass Spectra (SWATH-MS) that in the future might increase sensitivity and selectivity of the rtPA-associated sICH risk calculations.

There are obviously plenty other studies aiming at identifying serum or plasma prognostic biomarkers of rtPA-related hemorrhagic transformation in patients with acute ischemic stroke. However, in general they are based on far different methodological, technical and analytical approach to that described in this paper, and the selected biomarker—candidates have been characterized by relatively low sensitivity or selectivity thus so far not being applicable for clinical practice, and need further investigations^24–33^.

## Results

Careful visual assessment of the initial neuroimaging data revealed a DWI hyperintensive lesion corresponding with neurological deficit in 30 out of 41 patients (73%). The ICH was found in 9 cases on the 5–9 day follow up SWI and T2* MR Imaging. In this initial phase of the study we did not differentiate ICH into symptomatic and asymptomatic. However, the clinical status and neuroimaging characteristics of each individual participant are listed in the Supplementary Table 1. The summary of the clinical and neuroimaging characteristics of the participants divided into two groups—with and without ICH are presented in Table 2.

**Table 2:** Basic clinical and neuroimaging characteristics of ischemic stroke patients with and without intracranial hemorrhage after rtPA treatment.

	Without ICH (N = 32)	With ICH (N = 9)	*p* value*
**NIHSS on admission**			0.221
Min–Max	1.00–18.00	2.00–17.00	
Mean	6.12	7.78	
Median (Q1,Q3)	5.00 (3.00, 6.25)	7.00 (4.00, 11.00)	
**NIHSS on discharge**			0.458
Min–Max	0.00–15.00	0.00–7.00	
Mean	1.44	2.22	
Median (Q1,Q3)	0.50 (0.00, 1.25)	1.00 (0.00, 3.00)	
**Age**			0.344
Min–Max	19.00–94.00	51.00–91.00	
Mean	64.56	72.56	
Median (Q1,Q3)	67.00 (52.00, 80.25)	71.00 (59.00, 84.00)	
**Sex, male**	11 (34.4%)	6 (66.7%)	0.128
**Hypertension**	23 (71.9%)	8 (88.9%)	0.410
**Diabetes**	8 (25.0%)	3 (33.3%)	0.680
**Hiperlipidemia**	20 (62.5%)	8 (88.9%)	0.228
**Atrial Fibrillation**	7 (21.9%)	0 (0.0%)	0.315
**Active smoking**	10 (31.2%)	3 (33.3%)	< 1.000
**Baseline infarct volume [ml]**			0.050
Min–Max	0.00–64.02	1.14–55.57	
Mean	11.88	23.87	
Median (Q1,Q3)	2.63 (0.00, 17.02)	16.02 (10.36, 36.91)	

ICH—intracranial hemorrhage, NIHSS—National Institute of Health Stroke Scale; Q1—the first quartile; Q3—the third quartile; *Mann–Whitney U test was applied for comparison of quantitative data, whereas Fisher’s exact test was used to compare binomial data; *p* value equal or less than 0.05 was considered statistically significant.

We have identified 261 proteins at 1% FDR in a joint database search of unfractionated and fractionated plasma and serum pool samples of ischemic stroke patients (Supplementary Tables 2, 2a and 2b). In the case of 237 of these, 2 or more confidently recognized peptides were detected. With the use of this database search as a spectral library for SWATH-MS analysis of clinical samples, 180 proteins could be reliably quantified in a relative manner. DIA measurements of plasma and serum samples were processed simultaneously, and obtained results were divided into two sets of data prior to the statistical analysis.

Patients were assigned to the test group (i.e.with ICH, n = 9) and the control group (without ICH, n = 32) resulting in two experimental datasets subjected to statistical analysis by Mann–Whitney U tests. Any changes in protein concentrations with *p* value < 0.05 were considered significant, no matter the extent of the concentration change. In all investigated statistical comparisons, we detected 15 differential proteins (Table 3) in both analyzed fluids. Eight of them were found in serum as specific to this biological fluid, (2 proteins present at higher concentrations and 6 proteins present at lower concentrations in the test group). Immunoglobulin heavy constant gamma 4 was present at the most increased concentrations in the test group (about 3.6-fold) and thyroxine-binding globulin was present at the most decreased concentrations (almost 0.6-fold). In case of plasma, 7 proteins were present at different concentrations with *p* value < 0.05. All seven proteins were present at higher concentrations in the test group with immunoglobulin heavy variable 5–51 demonstrating highest increase (more than 1.7-fold). Notably, fibrinogen beta and gamma chains were present in this group with 1.3-fold increase.

**Table 3:** Serum and plasma protein candidates for biomarkers related to the intracranial hemorrhage after rtPA treatment in ischemic stroke patients.

Uniprot ID	Protein name	Serum			Plasma	
		*p* value*	Fold change†		*p* value*	Fold change†
Q96PD5	N-acetylmuramoyl-L-alanine amidase	**0.005**		**0.804**	0.115	1.138
P08697	Alpha-2-antiplasmin	**0.008**		**0.616**	0.614	0.935
P02768	Serum albumin	**0.009**		**1.372**	0.682	1.038
P05543	Thyroxine-binding-globulin	**0.011**		**0.598**	0.592	1.158
P02790	Hemopexin	**0.015**		**0.676**	0.614	0.994
P01861	Immunoglobulin heavy constant gamma 4	**0.024**		**3.618**	0.950	1.056
P01019	Angiotensinogen	**0.037**		**0.747**	0.508	0.980
P02749	Beta-2-glycoprotein 1	**0.044**		**0.718**	0.413	1.118
P04278	Sex hormone-binding globulin	0.889		1.002	**0.006**	**1.483**
P02675	Fibrinogen beta chain	0.313		0.760	**0.017**	**1.377**
P02679	Fibrinogen gamma chain	0.164		0.765	**0.021**	**1.230**
A0A0C4DH38	Immunoglobulin heavy variable-5–51	0.999		1.106	**0.021**	**1.779**
Q06033	Inter-alpha-trypsin inhibitor heavy chain H3	0.297		0.852	**0.025**	**1.213**
P02649	Apolipoprotein E	0.487		1.105	**0.027**	**1.532**
P36955	Pigment epithelium-derived factor	0.651		0.909	**0.038**	**1.270**

^†^The fold change represents the change of protein concentration between the patients with and without ICH, *Any changes in protein concentrations with *p* value < 0.05 were considered significant, no matter the extent of the concentration change.

**Figure 1:**
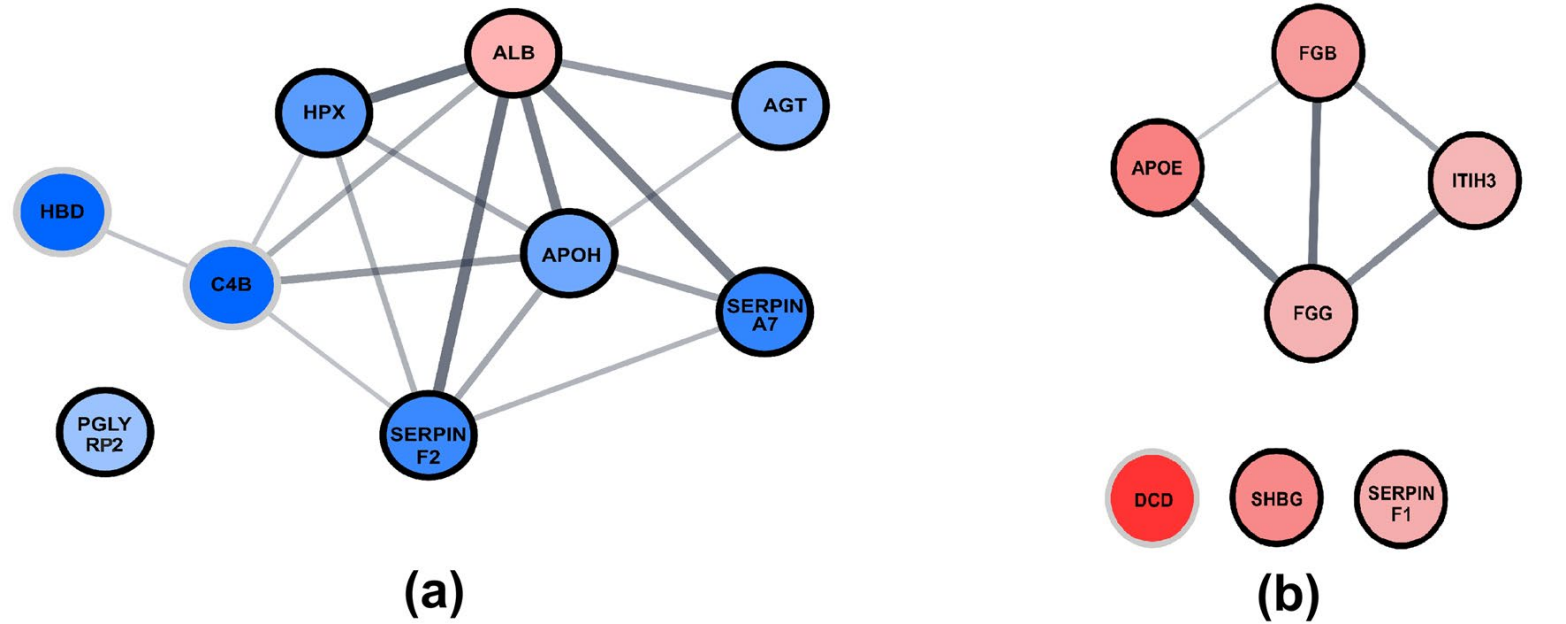
The Cytoscape visualization of STRING-generated network composed of experimentally verified protein–protein interactions among the quantified proteins (**a**) for serum and (**b**) for plasma. Nodes with a bold edge correspond to proteins with statistically significant change between the groups (*p* value < 0.05), whereas nodes with grey edges correspond to proteins with a verified interaction with those proteins and at least twofold concentration change between the groups, which was however insignificant (*p* value > 0.05). The gradation of the fill corresponds to the value of the concentration fold changes, the darker the greater the change, shades of red and blue correspond to increased and decreased concentration in the test group, respectively.

To get a more thorough look at the proteomic landscape of our patients with ICH, we constructed interaction networks in Cytoscape software and conducted basic functional enrichment analysis. In these networks, we included also the proteins with at least twofold concentration change, which interacted well with those described previously (Fig. 1). The network constructed for serum was based on 7 differentiators recognized by STRING database, with 6 proteins forming the network along with 2 additional proteins. We conducted functional enrichment analysis on constructed network in terms of GO Process, which yielded terms associated with regulation of response to stress and to external stimulus (APOH, HPX, SERPINF2, PGLYRP2, AGT, C4B), negative regulation of endopeptidase activity (SERPINF2, SERPINA7, AGT, C4B), and regulation of blood vessel diameter by renin-angiotensin (SERPINF2, AGT). Moreover, top three Reactome Pathways terms were: response to elevated platelet cytosolic Ca^2+^, platelet degranulation (APOH, ALB, SERPINF2), and scavenging of heme from plasma (HPX, ALB). The network for plasma was based on 6 differentiating proteins, out of which 4 interacted only with each other. Dermcidin, present at more than twofold higher concentration in the test group, was added to the network. Primarily enriched GO Processes included: negative regulation of response to external stimulus (APOE, SERPINF1, FGB, FGG), negative regulation of blood coagulation and regulation of blood vessel diameter (APOE, FGB, FGG). The main enriched Reactome pathways consisted of: integrin signaling, platelet aggregation (FGB, FGG), and response to elevated platelet cytosolic Ca^2+^ (FGB, FGG, ITIH3).

## Discussion

In this pilot study we demonstrate that pre-treatment blood proteomic profiles of ischemic stroke patients differ depending on whether the patients develop rtPA-associated ICH or not. SWATH-MS quantification of plasma and serum proteins might allow for robust selection of blood biomarkers to increase the prospective assessment of rtPA-associated ICH, and it is likely that these parameters will strengthen assessments of the brain bleeding risk over that based solely on clinical and neuroimaging characteristics.

Taking a small sample analyzed for this paper, any major pathophysiological conclusions related to individual compounds might be too speculative. Moreover, due to the same reason, we did not analyze these pilot data with the main endpoint division of patients i.e. into those with symptomatic versus asymptomatic ICH, working on any versus non-ICH instead. These limitations must be taken into account in interpreting these results, whereas the portion of the pathophysiological discussion below might be only treated as selective and exemplary.

Ning et al. found that rtPA treated patients have distinct degradomic pattern from those untreated at the early (< 24 h post-tPA) phase^22^. Their analysis of the individual degradomic fractions revealed degradation of fibrinogen and alpha 2 macroglobulin, which are thrombolysis pathway-related proteins. They were able to identify different components of fibrinogen subunits in the post-thrombolytic profiles (alpha and gamma subunits). Our data reveal increase (about 1.3-fold) of the fibrinogen beta and gamma chain in rtPA-associated ICH, in comparison with non-ICH patients, which might be partially consistent with the former despite different design of both studies with pre- versus post-thrombolysis blood sample analyses. Although the latter do not allow for direct comparisons of our report with Dagonnier et al., it is worth noting that this study also indicated substantial changes in fibrinogen serum concentrations during the first 24 h after stroke^23^.

SWATH, the proteomic analysis used in this study, allows only to determine the trend of changes, not the actual protein concentration, but its sensitivity and selectivity is sufficient to distinguish between patient groups according to the endpoint characteristics. Moreover, the micro LC system with QT of spectrometer seems a reasonable solution for clinical studies even in emergency settings because, compared to nano LC systems, the time of analysis is significantly reduced from hours to several dozen minutes and may be even shorter with future technical developments. It also provides the ability to maintain a high throughput in opposition to nano systems, which makes it possible to test a large number of samples in a relatively short time with low operator intervention in maintaining the system in continuous operation.

Rapid and comprehensive clinical assessment is crucial to improve functional outcome and reduce mortality. Stroke centers, in order to ensure short door-to-needle time, use CT scan as a basic neuroimaging modality in acute ischemic stroke prior to rtPA administration. In our study, we applied baseline MRI, with “GO-Brain”, fast, early phase, stroke-dedicated, Siemens-designed protocol that comprises only necessary sequences and lasts only a few minutes more than regular head CT. Nevertheless, this protocol imposes considerable pressure on stroke neurologists, nurses, paramedics and radiologists, that need to act even faster in order to prepare patients for the rtPA treatment in the shortest possible time. The brisk and efficient cooperation of emergency department staff is indispensable to complete essential steps in qualification process—blood samples collection and brain MRI scan prior to rtPA administration, but also in the follow-up phase 7–9 days after the incident of stroke. Altogether, this challenging and unique approach represents major asset of the project and adds additional value to its results.

This project aims at selection of molecular, neuroimaging and clinical parameters combined to mathematically support pre-treatment estimation of the risk of rtPA-associated ICH. Development of a new tool for selection of patients at risk will eventually require ‘big data’ analysis (350–400 patients, multiple parameters of various origin for each) with deep learning techniques. However, in this paper, based on a pilot patient sample and analyzed with simple statistical approach, we only demonstrate that information on selected blood protein and peptide concentrations might potentially increase the power of the risk-calculation engine over that based solely on clinical and neuroimaging characteristics. In our analysis, the results of statistical calculations do not contain *p* values adjustment for multiple testing; we prespecified and designed this pilot study to show and discuss a special, hypothesis free approach in clinical medicine, whereas the original portion of this study is primarily exploratory, with no intention to confirm the usefulness of any biomarker pattern at this stage of the major project.

## Materials

### Study population and blood collection

All study participants were acute ischemic stroke patients hospitalized at the Stroke Unit of the Department of Adult Neurology, University Clinical Center, Medical University of Gdansk, Poland, between March 20, 2019 and February 20, 2020.

During the pilot recruitment period we performed 94-MRI examinations in patients with suspected acute stroke. It is worth noting that—to avoid any treatment delays—we used only a rapid MR protocol that includes only a few sequences necessary to gain neuroimaging information on potential contraindications to systemic thrombolysis (the “GoBrain” application). Fifty-six patients received i.v. rtPA treatment according to standard inclusion and exclusion criteria based on major management guidelines. The only additional inclusion criterion was lack of contraindication to MRI. All patients underwent MRI and blood sampling prior to the treatment, and on day 7–9 from stroke onset. Following this, we have excluded 15 patients due to lack- or poor quality of follow-up neuroimaging data or lack of follow-up blood sample. 41 patients entered the final analysis.

For the initial (pilot) phase of the study, we enrolled 41 patients. The study was approved by the local ethics committee: The Independent Bioethics Committee for Scientific Research at the Medical University of Gdańsk, Poland. All research procedures were performed in accordance with the principles of the World Medical Association Declaration of Helsinki. All the patients provided informed consent for the involvement in the study. Comprehensive clinical data on the study group was gathered. The summary clinical and neuroimaging characteristics of the participants are presented in Table 2. The clinical status and neuroimaging characteristics of each individual participant are listed in the Supplementary Table 1.

Peripheral venous blood samples were collected on admission, just before the rtPA treatment. Each patient had two blood samples collected—a EDTA-coated tube to acquire plasma and one with silica clot activator to obtain serum (Becton Dickinson Vacutainer). Shortly after collection samples were cetrifuged, serum and plasma were transferred to Eppendorf tubes and were safely stored in deep-freeze (− 80 °C). The collection of serum and plasma samples was then sent to the Intercollegiate Faculty of Biotechnology of University of Gdansk and Medical University of Gdansk, Poland, for proteomic analysis.

### Neuroimaging studies

All study participants had MRI scan on admission and a non-contrast CT scan the next day (day 2) after the rtPA treatment. Additionally, 39 patients had MRI scan on day 5–9 after admission.

All MRI examinations were performed using 1.5T Siemens Magnetom Aera system. First MRI examination was performed using fast “GO-Brain” protocol composed of the following sequences: sagittal T1 weighted, axial diffusion-weighted imaging (DWI) (b value 0 and 800), and ADC maps, axial T2 weighted fluid attenuation inversion recovery (FLAIR) and axial T2* weighted. The second MRI protocol included the same sequences as the initial MRI, together with additional sequences: axial diffusion tensor imaging (DTI), axial susceptibility-weighted imaging (SWI), three-dimensional axial T1 weighted, and sagittal T2 weighted sequences.

The short MRI protocol sequences including DWI with ADC maps, and FLAIR were assessed by an experienced radiologist.Volumes of the lesions corresponding to acute ischemia were calculated based on visual assessments of their expansion using the software (syngo.via) integrated with the machine console (see Supplementary Table 1).

The ICH presence and expansion was assessed with 5–9 day SWI and T2*-weighted MR Imaging. In cases where follow up MRI scan wasn’t available (2 patients) the assessment was based on the routine post-rtPA follow-up CT scan (see Supplementary Table 1).

### Proteomics

#### Protein fractionation for spectral library preparation

Pool samples of serum and plasma were fractionated prior proteolytic digestion to enrich the spectral library employed in SWATH-MS quantification. Three strategies were applied: (i) ultrafiltration, (ii) immunodepletion, and (iii) protein enrichment. Ultrafiltration was conducted using Amicon filters (Merck) with membranes of 30 kDa and 100 kDa molecular weight cutoff. Both retentate and filtrate fractions were analyzed. Immunodepletion involved application of the Multiple Affinity Removal Spin Cartridge Human 14 (MARS-14) kit (Agilent Technologies, Santa Clara, CA) according to manufacturer's protocol. Besides the isolated low abundant protein fractions, the bound high abundant protein fractions were also further processed for MS measurements. Protein enrichment was carried out with the ProteoMiner kit (BioRad) according to the manufacturer's protocol.

All serum and plasma samples were prepared for proteomic analysis according to standard Filter Aided Sample Preparation (FASP) procedure^34^. 50 µl of the sample was mixed with 200 µl of the solution containing 1% SDS, 100 mM Tris/HCl pH 8, 50 mM DTT (lysis solution) and incubated at 95 °C for 10 min. First, the protein concentration was established for each sample by measuring absorbance at 280 nm (MultiskanTM Thermo) using the µDrop plate. For each digestion in triplicate, 100 µg of proteins were added to 200 ml of 8 M urea in 0.1 M Tris/HCl pH 8.5 (UA solution) placed in the 10 kDa microcons (Merck) and centrifuged at 10,000×*g* for 30 min. Washing and centrifuging steps with UA were repeated 3 times for samples were protein concentration was higher than 10 mg/ml; in the case of lower content, two additional washing steps with UA were added. Next, 100 µl of 55 mM iodoacetamide (IAA) solution in UA was added, and samples were kept in the dark for 20 min. After centrifugation, alkylated proteins were washed on the membrane three times with 100 µl of UA solution and two times with 100 µl of 50 mM Tris HCl pH 8.5 buffer (DB). Finally, the microcones were placed in new tubes, and a solution containing 2 µg of trypsin was added to each sample. The digestion was performed overnight at 37 °C in the incubation chamber. Tryptic peptides were collected after centrifugation at 10,000×*g* for 20 min. Each membrane was additionally washed with 125 and 100 µl of DB solution. Before the final clean-up, the peptide concentration was measured. Generated tryptic peptides were desalted with StageTips according to the protocol described by Rappsilber et al.^35^. For each desalting step, 10 µg of the peptides was taken and desalted on StageTip containing three layers of 3 M Empore C18 exchange disks. Peptides eluated by 100 µl 60% acetonitrile/1% acetic acid were concentrated to 20 µl prior to MS analysis.

#### Liquid chromatography and mass spectrometry

LC–MS/MS analysis was performed with the use of a Triple TOF 5600 + mass spectrometer (SCIEX Framingham, MA) coupled with the Ekspert MicroLC 200 Plus System (Eksigent, Redwood City, CA, USA). All chromatographic separations were performed on the ChromXP C18CL column (3 µm, 120 Å, 150 × 0.3 mm). The chromatographic gradient for each MS run was 8–40% B (solvent A 0% aqueous solution 0.1% formic acid, solvent B 100% acetonitrile 0.1% formic acid) in 30 min. The whole system was controlled by the SCIEX Analyst TF 1.7.1 software. Measurements for spectral library were acquired in triplicate. Each cycle of applied DDA method comprised of precursor spectra accumulation in 250 ms in the range of 400–100 m/z followed by top 20 precursor’s product ion spectra accumulation in 100 ms in the range of 100–1500 mz, resulting in a total cycle time of 2.3 s. Formerly fragmented precursor ions were dynamically excluded.

#### SWATH mass spectrometry

Experiments on clinical samples were performed in a looped product ion mode with the spectrometer set to high sensitivity focus. A set of 25 transmission windows of variable width was constructed with the use of SwathTUNER software based on equalized frequency of precursor ions and covered the precursor mass range of 400–1000 m/z^36^. The collision energy for each window was calculated for + 2 to + 5 charged ions centered upon the window with a spread of five. The SWATH-MS survey scan was acquired in the range covered by constructed windows in the beginning of each cycle with the accumulation time of 50 ms, and following SWATH-MS/MS spectra product ion scans were collected in the range of 100 to 1500 m/z in 40 ms, which resulted in the total cycle time of 1.1 s.

#### Data analysis

Database search was performed with ProteinPilot 4.5 software (Sciex) using the Paragon algorithm against the SwisProt Homo sapiens database (version from 8.04.20, 20 350 entries) with an automated false discovery rate, and standard parameters (alkylation of cysteine residues by iodoacetamide, digestion by trypsin, ID focus on biological modifications).

Next, a spectral library was created with the group file data processing in PeakView v. 2.2 (SCIEX), with settings as described in detail by Lewandowska et al.^37–39^. Joint search for library generation included unfractionated pool samples and samples fractionated as described in “Protein fractionation for spectral library preparation” paragraph, both serum and plasma. Files from SWATH experiments for each sample were downloaded to PeakView software and processed with the previously established library. Resulting data were exported to the .xml file and exported to Marker View software. All data were normalized using total area sums (TAS) approach. The mass spectrometry proteomics data have been deposited to the ProteomeXchange Consortium via the PRIDE partner repository with the dataset identifier PXD021713^40^. Cytoscape 3.8.0^41^ and STRING 11.0^42^ were used for the interactome network visualization.

### Statistical analysis

Continuous variables were described as medians and the first and third quartiles. The technical variability of the quantitative proteomics was calculated using the quartile coefficient of dispersion. Categorical data was presented as frequencies and percentages. We applied the Mann–Whitney U test for comparison of quantitative variables between unpaired two samples of patients—with and without ICH, whereas the Fisher exact test was used to compare binomial data. The 2-tailed tests were carried out at a significance level of *p* ≤ 0.05. All statistical analyses were performed using the R statistical package (version 3.6.3; https://www.r-project.org/).

## Conclusions

This study is the first to show pre-treatment characteristics of blood protein profiles for haemorrhagic complications related to intravenous administration of rtPA in acute ischemic stroke patients.

However, the biomarker list will require confirmation with a larger sample (in course) and in other populations of stroke patients. This project aims at section of new parameters to mathematically support pre-treatment estimation of the risk of rtPA-associated ICH but further considerations of biological roles and relations between the selected biomarkers might be also of special interest.

The main general target of this paper is to indicate an extensive significance of combined mathematical analysis of data (or big data) of different origin (proteomic, other “omic”, demographic, clinical, imaging-neuroimaging, environmental) in clinical medicine to generate tools that might support individual therapeutic decisions throughout precise calculation of probability of various outcome scenarios.

## Supplementary Information

Supplementary Table 1.

Supplementary Table 2.

Supplementary Table 3.

Supplementary Table 4.
