# Supplementary material for: SWATH-MS for prospective identification of protein blood biomarkers of rtPA-associated intracranial hemorrhage in acute ischemic stroke: a pilot study
Source: Sci Rep. 2021 Sep 21;11:18765. doi: 10.1038/s41598-021-97710-9 (PMC8455557; doi:10.1038/s41598-021-97710-9)
Supplement: Supplementary file 3 — Supplementary Table 3. [file 41598_2021_97710_MOESM3_ESM.pdf]

SWATH- MS for prospective identification of protein blood biomarkers of rtPA-associated intracranial hemorrhage in acute ischemic stroke: a pilot study

Bartosz Karaszewski, Anna Gójska-Grymajło, Paulina Czaplewska, Bartosz Jabłoński, Aleksandra E. Lewandowska, Daria Ossowska, Adam Wyszomirski, Marek Hałas, Edyta Szurowska

Supplementary Table 2a. *Differences in distributions of* ***serum*** *proteins in patients with and without intracranial hemorrhage (ICH).*

	Without ICH (N=31)	With ICH (N=8)	p value
**Q96PD5 (N-acetylmuramoyl-L-alanine amidase)**			0.005
Min-Max	80530 - 231020	81564 - 125403	
Mean	134707	105072	
Median (Q1,Q3)	132403 (112506, 144959)	106430 (97185, 112539)	
Quartile coefficient of dispersion	0.13	0.07	
**P08697 (Alpha-2-antiplasmin)**			0.008
Min-Max	83714 - 371646	81827 - 234129	
Mean	196150	132496	
Median (Q1,Q3)	187178 (159237, 248123)	115280 (104682, 147788)	
Quartile coefficient of dispersion	0.22	0.17	
**P02768 (Serum albumin)**			0.009
Min-Max	15938112 - 70009459	40556735 - 87367623	
Mean	46707880	63506160	

Median (Q1,Q3)	45342383 (39620003, 55110740)	62189923 (57234653, 67960436)	
Quartile coefficient of dispersion	0.16	0.09	
**P05543 (Thyroxine-binding globulin)**			0.011
Min-Max	88729 - 744012	87469 - 270867	
Mean	246129	150981	
Median (Q1,Q3)	228327 (170936, 283297)	136446 (126292, 162640)	
Quartile coefficient of dispersion	0.25	0.13	
**P02790 (Hemopexin)**			0.015
Min-Max	1529212 - 10034154	2103865 - 7912770	
Mean	7057868	5085098	
Median (Q1,Q3)	7305937 (5506160, 8763109)	4938764 (3924808, 6221529)	
Quartile coefficient of dispersion	0.23	0.23	
**P01861 (Immunoglobulin heavy constant gamma 4)**			0.024
Min-Max	18771 - 894352	113538 - 840738	
Mean	233105	472844	
Median (Q1,Q3)	134459 (69795, 346066)	486461 (272433, 685669)	
Quartile coefficient of dispersion	0.66	0.43	
**P01019 (Angiotensinogen)**			0.037
Min-Max	137709 - 1159487	147931 - 682099	
Mean	677010	478946	

Median (Q1,Q3)	686761 (519703, 871019)	512997 (352909, 662496)	
Quartile coefficient of dispersion	0.25	0.3	
**P02749 (Beta-2-glycoprotein 1)**			0.044
Min-Max	56509 - 1169304	201327 - 464766	
Mean	541356	348704	
Median (Q1,Q3)	488446 (355246, 760907)	350912 (261735, 446516)	
Quartile coefficient of dispersion	0.36	0.26	
**P19823 (Inter-alpha-trypsin inhibitor heavy chain H2)**			0.056
Min-Max	729341 - 3171013	834291 - 2068879	
Mean	1780560	1436200	
Median (Q1,Q3)	1767384 (1484983, 2005710)	1549345 (1319857, 1581386)	
Quartile coefficient of dispersion	0.15	0.09	
**P32119 (Peroxiredoxin-2)**			0.056
Min-Max	36012 - 766092	28774 - 127392	
Mean	141264	78912	
Median (Q1,Q3)	107451 (74532, 162120)	80482 (60335, 92560)	
Quartile coefficient of dispersion	0.37	0.21	
**Q14624 (Inter-alpha-trypsin inhibitor heavy chain H4)**			0.060
Min-Max	490176 - 4120163	568790 - 2437440	
Mean	2145604	1584043	
Median (Q1,Q3)	2204921 (1744378, 2431096)	1692654 (1143200, 2041087)	

Quartile coefficient of dispersion	0.16	0.28	
**P02787 (Serotransferrin)**			0.071
Min-Max	9847917 - 25900689	10361381 - 18522804	
Mean	17548875	14668587	
Median (Q1,Q3)	17617716 (15307978, 20187456)	14689258 (12383437, 16891429)	
Quartile coefficient of dispersion	0.14	0.15	
**P00915 (Carbonic anhydrase 1)**			0.071
Min-Max	32184 - 1097373	20227 - 110685	
Mean	207366	57991	
Median (Q1,Q3)	75716 (51485, 282951)	52932 (36028, 72638)	
Quartile coefficient of dispersion	0.69	0.34	
**P10909 (Clusterin)**			0.076
Min-Max	1264661 - 3996865	1018999 - 2779293	
Mean	2381176	1870060	
Median (Q1,Q3)	2271576 (1893574, 2799610)	1866170 (1487033, 2179787)	
Quartile coefficient of dispersion	0.19	0.19	
**P20742 (Pregnancy zone protein)**			0.076
Min-Max	7472 - 112104	10875 - 49231	
Mean	40409	24558	
Median (Q1,Q3)	32290 (23374, 42495)	18408 (15458, 31921)	
Quartile coefficient of dispersion	0.29	0.35	
**P01024 (Complement C3)**			0.082

Min-Max	1746065 - 11137908	1682627 - 8684874	
Mean	6979686	5226601	
Median (Q1,Q3)	7587452 (4736200, 8491171)	5368823 (4129150, 6103989)	
Quartile coefficient of dispersion	0.28	0.19	
**P00450 (Ceruloplasmin)**			0.088
Min-Max	953846 - 3575269	1203621 - 2590327	
Mean	2553162	2132529	
Median (Q1,Q3)	2560285 (2166887, 2936375)	2235804 (1968824, 2463766)	
Quartile coefficient of dispersion	0.15	0.11	
**P09871 (Complement C1s subcomponent)**			0.088
Min-Max	91828 - 427834	105661 - 277521	
Mean	220083	171761	
Median (Q1,Q3)	203423 (177966, 253096)	169370 (149330, 184900)	
Quartile coefficient of dispersion	0.17	0.11	
**P02654 (Apolipoprotein C-I)**			0.088
Min-Max	388454 - 2864062	470080 - 1550752	
Mean	1563911	1131136	
Median (Q1,Q3)	1489515 (1144457, 2042632)	1245277 (977319, 1348898)	
Quartile coefficient of dispersion	0.28	0.16	
**P19652 (Alpha-1-acid glycoprotein 2)**			0.088
Min-Max	996578 - 4198103	1240005 - 1613404	

Mean	1959560	1442926	
Median (Q1,Q3)	1733767 (1412742, 2398621)	1444711 (1364402, 1533956)	
Quartile coefficient of dispersion	0.26	0.06	
**P19827 (Inter-alpha-trypsin inhibitor heavy chain H1)**			0.095
Min-Max	596883 - 3094317	572017 - 1610412	
Mean	1523548	1150994	
Median (Q1,Q3)	1461853 (1046430, 1852136)	1263532 (1017523, 1337547)	
Quartile coefficient of dispersion	0.28	0.14	
**P04004 (Vitronectin)**			0.095
Min-Max	1301529 - 5187862	1048719 - 3915051	
Mean	2971785	2388592	
Median (Q1,Q3)	2925887 (2390483, 3408604)	2173433 (1995989, 2912911)	
Quartile coefficient of dispersion	0.18	0.19	
**P25311 (Zinc-alpha-2-glycoprotein)**			0.102
Min-Max	649967 - 2533847	1138458 - 2213197	
Mean	1273333	1505380	
Median (Q1,Q3)	1286002 (1090644, 1414985)	1349343 (1320890, 1592671)	
Quartile coefficient of dispersion	0.13	0.09	
**P00742 (Coagulation factor X)**			0.102
Min-Max	32917 - 142858	48080 - 72085	
Mean	77407	59015	

Median (Q1,Q3)	71622 (57815, 92595)	58534 (49988, 66327)	
Quartile coefficient of dispersion	0.23	0.14	
**P68871 (Hemoglobin subunit beta)**			0.110
Min-Max	175440 - 31066896	232399 - 4707689	
Mean	6481595	1694692	
Median (Q1,Q3)	1887353 (857031, 9445696)	1452066 (387875, 2149624)	
Quartile coefficient of dispersion	0.83	0.69	
**P05546 (Heparin cofactor 2)**			0.110
Min-Max	304463 - 1899776	389390 - 1423889	
Mean	1042653	820499	
Median (Q1,Q3)	1079588 (716714, 1280902)	745858 (556622, 1053529)	
Quartile coefficient of dispersion	0.28	0.31	
**P07358 (Complement component C8 beta chain)**			0.110
Min-Max	80940 - 365631	69712 - 228656	
Mean	201374	155982	
Median (Q1,Q3)	218786 (134572, 241273)	142834 (120930, 203530)	
Quartile coefficient of dispersion	0.28	0.25	
**P02743 (Serum amyloid P-component)**			0.110
Min-Max	199820 - 914299	238512 - 721619	
Mean	571032	448394	
Median (Q1,Q3)	585780 (406572, 743827)	401083 (330162, 575279)	

Quartile coefficient of dispersion	0.29	0.27	
**O00391 (Sulfhydryl oxidase 1)**			0.110
Min-Max	12276 - 300422	13545 - 151610	
Mean	54275	35049	
Median (Q1,Q3)	26182 (19021, 78124)	18436 (16637, 22968)	
Quartile coefficient of dispersion	0.61	0.16	
**P02042 (Hemoglobin subunit delta)**			0.110
Min-Max	9151 - 822709	14856 - 86703	
Mean	180900	48361	
Median (Q1,Q3)	105702 (32979, 226254)	46337 (31139, 65960)	
Quartile coefficient of dispersion	0.75	0.36	
**P01008 (Antithrombin-III)**			0.118
Min-Max	508634 - 1859548	519105 - 1624816	
Mean	1281486	1022972	
Median (Q1,Q3)	1307321 (895972, 1677777)	1018850 (603030, 1339527)	
Quartile coefficient of dispersion	0.3	0.38	
**P01871 (Immunoglobulin heavy constant mu)**			0.118
Min-Max	925993 - 8721681	1369985 - 4483848	
Mean	3759780	2652999	
Median (Q1,Q3)	3404702 (2290810, 4898033)	2717607 (2029429, 3021727)	
Quartile coefficient of dispersion	0.36	0.2	
**P05156 (Complement factor I)**			0.118

Min-Max	130369 - 631258	158557 - 426556	
Mean	362631	283979	
Median (Q1,Q3)	342273 (244783, 441818)	276037 (199689, 348525)	
Quartile coefficient of dispersion	0.29	0.27	
**P08603 (Complement factor H)**			0.126
Min-Max	710494 - 1996111	552486 - 1144871	
Mean	1099158	924732	
Median (Q1,Q3)	1067216 (898353, 1220586)	930414 (864092, 1056757)	
Quartile coefficient of dispersion	0.15	0.1	
**P01042 (Kininogen-1)**			0.126
Min-Max	1183463 - 3533273	1418544 - 2804195	
Mean	2528164	2178966	
Median (Q1,Q3)	2609633 (2049227, 3001365)	2229574 (1882781, 2528366)	
Quartile coefficient of dispersion	0.19	0.15	
**P02652 (Apolipoprotein A-II)**			0.144
Min-Max	1063845 - 6743155	2871098 - 5625528	
Mean	3303624	3884846	
Median (Q1,Q3)	3025588 (2265192, 4108029)	3382875 (3062915, 4886732)	
Quartile coefficient of dispersion	0.29	0.23	
**P69905 (Hemoglobin subunit alpha)**			0.144
Min-Max	296171 - 22828327	324713 - 4413238	

Mean	5556441	2056674	
Median (Q1,Q3)	2556619 (1227559, 8941966)	1990975 (585750, 2835821)	
Quartile coefficient of dispersion	0.76	0.66	
**P02679 (Fibrinogen gamma chain)**			0.164
Min-Max	32923 - 287331	39305 - 93616	
Mean	91894	60991	
Median (Q1,Q3)	76462 (54605, 95301)	58469 (50508, 69185)	
Quartile coefficient of dispersion	0.27	0.16	
**P13645 (Keratin, type I cytoskeletal 10)**			0.164
Min-Max	36136 - 503761	28923 - 625327	
Mean	211077	341704	
Median (Q1,Q3)	205579 (107312, 297895)	385301 (165536, 509230)	
Quartile coefficient of dispersion	0.47	0.51	
**P01034 (Cystatin-C)**			0.164
Min-Max	5591 - 72790	13665 - 67434	
Mean	23370	30939	
Median (Q1,Q3)	20200 (15395, 23790)	25841 (19694, 36556)	
Quartile coefficient of dispersion	0.21	0.3	
**P02774 (Vitamin D-binding protein)**			0.175
Min-Max	1201721 - 3325668	1234349 - 2357953	
Mean	2003885	1707655	
Median (Q1,Q3)	1866633 (1589260, 2420938)	1594873 (1436519, 1975716)	

Quartile coefficient of dispersion	0.21	0.16	
**P00748 (Coagulation factor XII)**			0.175
Min-Max	48483 - 734964	88008 - 194082	
Mean	187935	137917	
Median (Q1,Q3)	163014 (136803, 219763)	138706 (102130, 165874)	
Quartile coefficient of dispersion	0.23	0.24	
**A0A0C4DH68 (Immunoglobulin kappa variable 2-24)**			0.175
Min-Max	55703 - 577342	118700 - 174307	
Mean	144038	146728	
Median (Q1,Q3)	119496 (94083, 160417)	143107 (130773, 165199)	
Quartile coefficient of dispersion	0.26	0.12	
**P02538 (Keratin, type II cytoskeletal 6A)**			0.186
Min-Max	36901 - 109000	27962 - 104099	
Mean	66105	54876	
Median (Q1,Q3)	61783 (52567, 79880)	49284 (37375, 66647)	
Quartile coefficient of dispersion	0.21	0.28	
**Q9Y5Y7 (Lymphatic vessel endothelial hyaluronic acid receptor 1)**			0.198
Min-Max	7723 - 51109	10104 - 25086	
Mean	22245	16510	
Median (Q1,Q3)	19912 (16797, 27823)	15257 (10693, 22429)	
Quartile coefficient of dispersion	0.25	0.35	
**P01031 (Complement C5)**			0.211

Min-Max	259223 - 1056965	384816 - 790077	
Mean	568727	502579	
Median (Q1,Q3)	555791 (469347, 619004)	465460 (407536, 539396)	
Quartile coefficient of dispersion	0.14	0.14	
**P43652 (Afamin)**			0.211
Min-Max	188516 - 973473	263403 - 672068	
Mean	563649	487940	
Median (Q1,Q3)	585279 (447839, 676914)	530390 (372132, 575294)	
Quartile coefficient of dispersion	0.2	0.21	
**P06681 (Complement C2)**			0.211
Min-Max	159450 - 931182	156455 - 297253	
Mean	267471	210673	
Median (Q1,Q3)	230903 (202658, 260421)	211741 (188341, 220830)	
Quartile coefficient of dispersion	0.12	0.08	
**P02766 (Transthyretin)**			0.211
Min-Max	287418 - 5310922	609623 - 2468366	
Mean	1580041	1223749	
Median (Q1,Q3)	1276872 (1026463, 2076808)	956296 (866952, 1417409)	
Quartile coefficient of dispersion	0.34	0.24	
**Q08380 (Galectin-3-binding protein)**			0.211
Min-Max	31111 - 194737	26628 - 148775	

Mean	78785	64611	
Median (Q1,Q3)	69831 (54754, 85529)	58093 (48320, 61851)	
Quartile coefficient of dispersion	0.22	0.12	
**P17936 (Insulin-like growth factor-binding protein 3)**			0.211
Min-Max	11676 - 80692	17407 - 39702	
Mean	39038	31021	
Median (Q1,Q3)	37552 (24973, 47385)	33535 (27752, 35914)	
Quartile coefficient of dispersion	0.31	0.13	
**P10643 (Complement component C7)**			0.223
Min-Max	124514 - 765490	166191 - 309387	
Mean	307305	247263	
Median (Q1,Q3)	280741 (235752, 367975)	248344 (216679, 295790)	
Quartile coefficient of dispersion	0.22	0.15	
**P02760 (Protein AMBP)**			0.237
Min-Max	76414 - 878459	76817 - 1116262	
Mean	488325	610191	
Median (Q1,Q3)	474989 (272930, 693242)	594507 (539801, 701933)	
Quartile coefficient of dispersion	0.44	0.13	
**P04275 (von Willebrand factor)**			0.251
Min-Max	32888 - 263254	16864 - 111114	
Mean	87735	65911	
Median (Q1,Q3)	77960 (54621, 97625)	74063 (42489, 81204)	

Quartile coefficient of dispersion	0.28	0.31	
**P05090 (Apolipoprotein D)**			0.251
Min-Max	338171 - 1686498	488310 - 1070242	
Mean	736051	820940	
Median (Q1,Q3)	719305 (529754, 870852)	885159 (699958, 954142)	
Quartile coefficient of dispersion	0.24	0.15	
**P0C0L5 (Complement C4-B)**			0.251
Min-Max	36738 - 459702	31124 - 276160	
Mean	172087	124626	
Median (Q1,Q3)	194521 (75740, 232762)	94572 (72770, 176383)	
Quartile coefficient of dispersion	0.51	0.42	
**P15169 (Carboxypeptidase N catalytic chain)**			0.251
Min-Max	11755 - 381594	14520 - 57175	
Mean	54860	35493	
Median (Q1,Q3)	44930 (35023, 55400)	34670 (20418, 52280)	
Quartile coefficient of dispersion	0.23	0.44	
**P01860 (Immunoglobulin heavy constant gamma 3)**			0.251
Min-Max	47805 - 3471666	120386 - 3602621	
Mean	1047819	1407784	
Median (Q1,Q3)	811484 (462867, 1681779)	1206095 (831689, 1714008)	
Quartile coefficient of dispersion	0.57	0.35	
**P01876 (Immunoglobulin heavy constant alpha 1)**			0.266

Min-Max	146137 - 22886824	6948247 - 21664617	
Mean	8478667	10850065	
Median (Q1,Q3)	8745899 (6499732, 10233352)	9337267 (8609602, 10751933)	
Quartile coefficient of dispersion	0.22	0.11	
**P29622 (Kallistatin)**			0.281
Min-Max	87065 - 662480	96992 - 273268	
Mean	236640	189151	
Median (Q1,Q3)	217047 (177131, 267847)	198998 (158165, 220522)	
Quartile coefficient of dispersion	0.2	0.16	
**Q96IY4 (Carboxypeptidase B2)**			0.281
Min-Max	40633 - 135787	34210 - 113807	
Mean	71265	62992	
Median (Q1,Q3)	67548 (60543, 77936)	63233 (47013, 67762)	
Quartile coefficient of dispersion	0.13	0.18	
**Q06033 (Inter-alpha-trypsin inhibitor heavy chain H3)**			0.297
Min-Max	90921 - 1731505	152897 - 316337	
Mean	294490	223751	
Median (Q1,Q3)	246020 (203601, 287491)	209488 (183908, 254171)	
Quartile coefficient of dispersion	0.17	0.16	
**P18428 (Lipopolysaccharide-binding protein)**			0.297
Min-Max	63004 - 705125	72880 - 293668	
Mean	208531	164408	

Median (Q1,Q3)	177004 (144192, 214395)	143465 (120545, 200686)	
Quartile coefficient of dispersion	0.2	0.25	
**P02671 (Fibrinogen alpha chain)**			0.313
Min-Max	55420 - 634019	58309 - 123471	
Mean	131312	91311	
Median (Q1,Q3)	91963 (76930, 144273)	94576 (64881, 115410)	
Quartile coefficient of dispersion	0.3	0.28	
**P02675 (Fibrinogen beta chain)**			0.313
Min-Max	63289 - 347320	71380 - 192038	
Mean	141444	108917	
Median (Q1,Q3)	116020 (86432, 156544)	88183 (83546, 126580)	
Quartile coefficient of dispersion	0.29	0.2	
**P01834 (Immunoglobulin kappa constant)**			0.313
Min-Max	8039875 - 39711275	10704840 - 38034915	
Mean	22516663	25958345	
Median (Q1,Q3)	23851235 (15342169, 28685033)	26056836 (22248716, 31662261)	
Quartile coefficient of dispersion	0.3	0.17	
**P02775 (Platelet basic protein)**			0.313
Min-Max	74877 - 993155	340870 - 702108	
Mean	442630	478682	
Median (Q1,Q3)	380165 (307478, 527889)	438373 (388897, 520626)	
Quartile coefficient of dispersion	0.26	0.14	

**P00740 (Coagulation factor IX)**			0.313
Min-Max	3916 - 88285	6643 - 49522	
Mean	24670	25970	
Median (Q1,Q3)	21106 (12600, 28364)	27568 (19772, 29403)	
Quartile coefficient of dispersion	0.38	0.2	
**P0DJI8 (Serum amyloid A-1 protein)**			0.330
Min-Max	46666 - 717250	80711 - 415639	
Mean	211489	248812	
Median (Q1,Q3)	147793 (100259, 271366)	260180 (174084, 324518)	
Quartile coefficient of dispersion	0.46	0.3	
**O75636 (Ficolin-3)**			0.330
Min-Max	103544 - 481080	116464 - 201859	
Mean	179008	156931	
Median (Q1,Q3)	173722 (143988, 194378)	158814 (139136, 172102)	
Quartile coefficient of dispersion	0.15	0.11	
**Q15582 (Transforming growth factor-beta-induced protein ig-h3)**			0.330
Min-Max	10574 - 101942	14833 - 51792	
Mean	30206	32192	
Median (Q1,Q3)	26946 (19052, 33914)	32349 (28338, 36834)	
Quartile coefficient of dispersion	0.28	0.13	
**A0A0B4J2H0 (Immunoglobulin heavy variable 1-69D)**			0.330
Min-Max	39763 - 351067	54795 - 186209	
Mean	121886	92703	

Median (Q1,Q3)	112557 (69302, 136169)	77280 (67486, 103839)	
Quartile coefficient of dispersion	0.33	0.21	
**P00736 (Complement C1r subcomponent)**			0.348
Min-Max	159012 - 488690	169209 - 282187	
Mean	264107	231673	
Median (Q1,Q3)	252569 (220863, 300778)	235859 (202708, 259853)	
Quartile coefficient of dispersion	0.15	0.12	
**P08185 (Corticosteroid-binding globulin)**			0.348
Min-Max	150842 - 952794	155401 - 498071	
Mean	438999	363555	
Median (Q1,Q3)	420370 (349754, 493085)	382671 (308245, 467581)	
Quartile coefficient of dispersion	0.17	0.21	
**P04003 (C4b-binding protein alpha chain)**			0.366
Min-Max	222159 - 1639497	294021 - 1278791	
Mean	877348	752672	
Median (Q1,Q3)	737473 (550113, 1252470)	728581 (437897, 1015649)	
Quartile coefficient of dispersion	0.39	0.4	
**P03952 (Plasma kallikrein)**			0.366
Min-Max	91720 - 303213	97232 - 275717	
Mean	201726	180662	

Median (Q1,Q3)	204128 (155014, 256163)	176475 (155802, 217046)	
Quartile coefficient of dispersion	0.25	0.16	
**P07357 (Complement component C8 alpha chain)**			0.366
Min-Max	128337 - 628348	109476 - 354873	
Mean	234189	201661	
Median (Q1,Q3)	194888 (172844, 269502)	182685 (149321, 232125)	
Quartile coefficient of dispersion	0.22	0.22	
**P43251 (Biotinidase)**			0.366
Min-Max	36103 - 199200	49413 - 112687	
Mean	82666	73341	
Median (Q1,Q3)	74171 (61639, 98478)	64187 (55269, 85188)	
Quartile coefficient of dispersion	0.23	0.21	
**P07477 (Trypsin-1)**			0.366
Min-Max	19547 - 366899	11523 - 92154	
Mean	64433	43034	
Median (Q1,Q3)	44898 (31738, 76290)	38709 (28810, 52711)	
Quartile coefficient of dispersion	0.41	0.29	
**P02647 (Apolipoprotein A-I)**			0.385
Min-Max	20196120 - 51154626	22688896 - 48845735	
Mean	38185789	39660379	
Median (Q1,Q3)	38878276 (33016133, 43710606)	42312439 (37460404, 44510749)	
Quartile coefficient of dispersion	0.14	0.09	

**P01011 (Alpha-1-antichymotrypsin)**			0.385
Min-Max	634921 - 14266570	880311 - 5223684	
Mean	3319673	2535023	
Median (Q1,Q3)	3234318 (2185059, 4045015)	2194835 (1178295, 3566471)	
Quartile coefficient of dispersion	0.3	0.5	
**P00747 (Plasminogen)**			0.385
Min-Max	594580 - 2164693	569357 - 1259705	
Mean	1024346	909489	
Median (Q1,Q3)	986005 (829266, 1192674)	877998 (722639, 1109716)	
Quartile coefficient of dispersion	0.18	0.21	
**P07225 (Vitamin K-dependent protein S)**			0.385
Min-Max	112911 - 509863	214993 - 391218	
Mean	323423	300559	
Median (Q1,Q3)	344269 (257230, 386123)	312524 (252112, 343187)	
Quartile coefficient of dispersion	0.2	0.15	
**P01602 (Immunoglobulin kappa variable 1-5)**			0.404
Min-Max	35390 - 338419	51807 - 457874	
Mean	124642	179480	
Median (Q1,Q3)	92960 (64716, 159123)	129092 (82174, 200718)	
Quartile coefficient of dispersion	0.42	0.42	
**P04217 (Alpha-1B-glycoprotein)**			0.424

Min-Max	801928 - 2798543	1592396 - 2842390	
Mean	2044772	2161286	
Median (Q1,Q3)	1950036 (1743566, 2437535)	2094196 (1915989, 2356696)	
Quartile coefficient of dispersion	0.17	0.1	
**P01782 (Immunoglobulin heavy variable 3-9)**			0.424
Min-Max	15565 - 1225023	23607 - 86656	
Mean	95205	50373	
Median (Q1,Q3)	54647 (34354, 64532)	45420 (35527, 61118)	
Quartile coefficient of dispersion	0.31	0.26	
**P01859 (Immunoglobulin heavy constant gamma 2)**			0.444
Min-Max	1068510 - 4171201	1500891 - 3467956	
Mean	2528040	2758375	
Median (Q1,Q3)	2515997 (1801456, 3246819)	3047268 (2114571, 3376611)	
Quartile coefficient of dispersion	0.29	0.23	
**P05154 (Plasma serine protease inhibitor)**			0.444
Min-Max	83465 - 465865	57947 - 224940	
Mean	148929	127938	
Median (Q1,Q3)	132039 (106762, 163319)	111103 (90667, 167991)	
Quartile coefficient of dispersion	0.21	0.3	
**P02533 (Keratin, type I cytoskeletal 14)**			0.444

Min-Max	33543 - 2030448	71290 - 303749	
Mean	189057	126071	
Median (Q1,Q3)	87938 (66861, 128356)	87213 (80471, 145783)	
Quartile coefficient of dispersion	0.32	0.29	
**P35542 (Serum amyloid A-4 protein)**			0.444
Min-Max	119483 - 697620	196963 - 876983	
Mean	392229	463482	
Median (Q1,Q3)	383291 (313052, 443761)	441336 (314058, 561797)	
Quartile coefficient of dispersion	0.17	0.28	
**P09172 (Dopamine beta-hydroxylase)**			0.444
Min-Max	8088 - 119789	9881 - 49393	
Mean	33768	25883	
Median (Q1,Q3)	26743 (17994, 44559)	24623 (15081, 34032)	
Quartile coefficient of dispersion	0.42	0.39	
**P0C0L4 (Complement C4-A)**			0.465
Min-Max	129570 - 937236	146646 - 528941	
Mean	357039	300433	
Median (Q1,Q3)	329018 (226605, 431544)	291033 (173480, 384681)	
Quartile coefficient of dispersion	0.31	0.38	
**P02746 (Complement C1q subcomponent subunit B)**			0.465
Min-Max	85132 - 640739	111052 - 214810	
Mean	194900	162763	

Median (Q1,Q3)	169740 (157592, 203310)	168012 (146181, 180521)	
Quartile coefficient of dispersion	0.13	0.11	
**P05452 (Tetranectin)**			0.465
Min-Max	52877 - 268264	45333 - 128934	
Mean	103112	90199	
Median (Q1,Q3)	94524 (76950, 122140)	96357 (71704, 105364)	
Quartile coefficient of dispersion	0.23	0.19	
**P05155 (Plasma protease C1 inhibitor)**			0.487
Min-Max	1771406 - 4459707	1818112 - 3785215	
Mean	2987772	2775924	
Median (Q1,Q3)	2886636 (2570355, 3404009)	2741713 (2395823, 3180431)	
Quartile coefficient of dispersion	0.14	0.14	
**P02649 (Apolipoprotein E)**			0.487
Min-Max	374098 - 1412469	375876 - 1851705	
Mean	842278	984314	
Median (Q1,Q3)	849646 (636224, 935769)	938887 (522317, 1334961)	
Quartile coefficient of dispersion	0.19	0.44	
**P13671 (Complement component C6)**			0.487
Min-Max	168958 - 599193	167885 - 393608	
Mean	273848	278679	
Median (Q1,Q3)	233674 (206462, 315502)	257949 (235255, 339678)	

Quartile coefficient of dispersion	0.21	0.18	
**Q16610 (Extracellular matrix protein 1)**			0.487
Min-Max	25373 - 179609	24520 - 100910	
Mean	65617	55277	
Median (Q1,Q3)	62427 (43502, 73921)	50327 (42559, 66410)	
Quartile coefficient of dispersion	0.26	0.22	
**P11226 (Mannose-binding protein C)**			0.487
Min-Max	17530 - 278141	14590 - 81953	
Mean	56847	43769	
Median (Q1,Q3)	47029 (29652, 67715)	39745 (33543, 52110)	
Quartile coefficient of dispersion	0.39	0.22	
**P13796 (Plastin-2)**			0.487
Min-Max	7819 - 256459	20462 - 166094	
Mean	103078	78794	
Median (Q1,Q3)	96712 (30036, 148555)	64257 (56795, 89138)	
Quartile coefficient of dispersion	0.66	0.22	
**Q9NZP8 (Complement C1r subcomponent-like protein)**			0.487
Min-Max	40918 - 184790	43871 - 105692	
Mean	69684	61884	
Median (Q1,Q3)	58961 (52961, 74438)	56671 (45986, 69613)	
Quartile coefficient of dispersion	0.17	0.2	
**P04433 (Immunoglobulin kappa variable 3-11)**			0.487
Min-Max	18978 - 1173626	205161 - 1026666	

Mean	369094	426504	
Median (Q1,Q3)	288057 (212244, 398917)	297820 (254629, 535238)	
Quartile coefficient of dispersion	0.31	0.36	
**P02748 (Complement component C9)**			0.509
Min-Max	319168 - 1264618	505632 - 958407	
Mean	756733	787340	
Median (Q1,Q3)	711092 (536040, 970282)	825044 (721606, 928252)	
Quartile coefficient of dispersion	0.29	0.13	
**B9A064 (Immunoglobulin lambda-like polypeptide 5)**			0.509
Min-Max	27429 - 201194	27241 - 131289	
Mean	98471	84002	
Median (Q1,Q3)	90991 (67930, 129162)	90995 (49425, 115869)	
Quartile coefficient of dispersion	0.31	0.4	
**P35527 (Keratin, type I cytoskeletal 9)**			0.531
Min-Max	70320 - 612280	92853 - 208514	
Mean	177225	145061	
Median (Q1,Q3)	141456 (119723, 186353)	140198 (105855, 174429)	
Quartile coefficient of dispersion	0.22	0.24	
**P51884 (Lumican)**			0.554
Min-Max	150578 - 746721	194322 - 327967	
Mean	310637	260317	

Median (Q1,Q3)	276739 (235374, 329154)	278608 (212465, 293968)	
Quartile coefficient of dispersion	0.17	0.16	
**P02741 (C-reactive protein)**			0.554
Min-Max	35565 - 725464	41139 - 374919	
Mean	112383	103894	
Median (Q1,Q3)	81903 (53008, 113377)	60307 (49618, 97617)	
Quartile coefficient of dispersion	0.36	0.33	
**P00746 (Complement factor D)**			0.554
Min-Max	8126 - 89555	15305 - 75509	
Mean	31723	36366	
Median (Q1,Q3)	28004 (21611, 37895)	31582 (25295, 38887)	
Quartile coefficient of dispersion	0.27	0.21	
**P35908 (Keratin, type II cytoskeletal 2 epidermal)**			0.578
Min-Max	32913 - 169392	17669 - 113874	
Mean	77569	69947	
Median (Q1,Q3)	64380 (51041, 102476)	77965 (31087, 103967)	
Quartile coefficient of dispersion	0.34	0.54	
**P22792 (Carboxypeptidase N subunit 2)**			0.578
Min-Max	116183 - 585664	101435 - 258294	
Mean	222314	190005	
Median (Q1,Q3)	195112 (161635, 246759)	192193 (166026, 233352)	
Quartile coefficient of dispersion	0.21	0.17	

**P00918 (Carbonic anhydrase 2)**			0.578
Min-Max	39841 - 287217	37794 - 137360	
Mean	96484	77858	
Median (Q1,Q3)	78012 (58096, 110806)	76362 (57748, 93549)	
Quartile coefficient of dispersion	0.31	0.24	
**Q13790 (Apolipoprotein F)**			0.578
Min-Max	12133 - 99367	31645 - 79059	
Mean	42205	43909	
Median (Q1,Q3)	37362 (30152, 48538)	40431 (35068, 44148)	
Quartile coefficient of dispersion	0.23	0.11	
**A0A0C4DH72 (Immunoglobulin kappa variable 1-6)**			0.602
Min-Max	431205 - 2759576	533757 - 1304517	
Mean	864585	778750	
Median (Q1,Q3)	767208 (656595, 906804)	785130 (587266, 836153)	
Quartile coefficient of dispersion	0.16	0.17	
**P06312 (Immunoglobulin kappa variable 4-1)**			0.602
Min-Max	234885 - 1063203	448051 - 905751	
Mean	665059	631241	
Median (Q1,Q3)	693344 (522766, 776118)	656255 (460440, 719208)	
Quartile coefficient of dispersion	0.2	0.22	
**P01009 (Alpha-1-antitrypsin)**			0.626
Min-Max	9392159 - 24829680	9018343 - 20693821	

Mean	15327428	14587904	
Median (Q1,Q3)	14250216 (12332346, 18526295)	14539614 (11142141, 18266492)	
Quartile coefficient of dispersion	0.2	0.24	
**P35858 (Insulin-like growth factor-binding protein complex acid labile subunit)**			0.626
Min-Max	74362 - 276671	95596 - 219760	
Mean	142733	134863	
Median (Q1,Q3)	135630 (111806, 166392)	116058 (111420, 148244)	
Quartile coefficient of dispersion	0.2	0.14	
**P00738 (Haptoglobin)**			0.651
Min-Max	251846 - 34448537	7571100 - 20944367	
Mean	16350081	16349820	
Median (Q1,Q3)	15315407 (12123849, 19959912)	18720936 (13165966, 20568490)	
Quartile coefficient of dispersion	0.24	0.22	
**P02751 (Fibronectin)**			0.651
Min-Max	483182 - 1678461	629758 - 1137123	
Mean	900931	850336	
Median (Q1,Q3)	838391 (691514, 1066375)	743294 (707449, 1069727)	
Quartile coefficient of dispersion	0.21	0.2	
**P00751 (Complement factor B)**			0.651
Min-Max	959773 - 2702958	1193324 - 2675381	
Mean	1600289	1671997	

Median (Q1,Q3)	1424671 (1342659, 1838239)	1584788 (1356698, 1773308)	
Quartile coefficient of dispersion	0.16	0.13	
**P06727 (Apolipoprotein A-IV)**			0.651
Min-Max	615899 - 5865244	1112026 - 5385869	
Mean	2617391	2564683	
Median (Q1,Q3)	2398189 (1738784, 3366817)	2157578 (1340581, 3330124)	
Quartile coefficient of dispersion	0.32	0.43	
**P36955 (Pigment epithelium-derived factor)**			0.651
Min-Max	197511 - 538400	254191 - 518685	
Mean	356939	341629	
Median (Q1,Q3)	346881 (290393, 430661)	315234 (282909, 376958)	
Quartile coefficient of dispersion	0.19	0.14	
**P02745 (Complement C1q subcomponent subunit A)**			0.651
Min-Max	247170 - 975199	225548 - 488919	
Mean	426151	383270	
Median (Q1,Q3)	375360 (315514, 481227)	413687 (284669, 483515)	
Quartile coefficient of dispersion	0.21	0.26	
**A0A0B4J1V0 (Immunoglobulin heavy variable 3-15)**			0.651
Min-Max	238271 - 3370212	510358 - 1374789	
Mean	874114	816700	

Median (Q1,Q3)	838856 (680627, 996876)	800867 (689991, 869121)	
Quartile coefficient of dispersion	0.19	0.11	
**P0DOY3 (Immunoglobulin lambda constant 3)**			0.651
Min-Max	69289 - 310002	74840 - 299792	
Mean	143293	148587	
Median (Q1,Q3)	144249 (109857, 159187)	116186 (81954, 201395)	
Quartile coefficient of dispersion	0.18	0.42	
**P26927 (Hepatocyte growth factor-like protein)**			0.651
Min-Max	8673 - 32827	9671 - 29006	
Mean	19155	17676	
Median (Q1,Q3)	18565 (14079, 23677)	17642 (13922, 20496)	
Quartile coefficient of dispersion	0.25	0.19	
**P06396 (Gelsolin)**			0.676
Min-Max	255360 - 1012415	396582 - 836519	
Mean	537347	553675	
Median (Q1,Q3)	473893 (403805, 628710)	454954 (433818, 693133)	
Quartile coefficient of dispersion	0.22	0.23	
**Q9UK55 (Protein Z-dependent protease inhibitor)**			0.676
Min-Max	37696 - 192842	28664 - 110169	
Mean	70026	61936	
Median (Q1,Q3)	65737 (54001, 76626)	65252 (37600, 77051)	

Quartile coefficient of dispersion	0.17	0.34	
**P01721 (Immunoglobulin lambda variable 6-57)**			0.676
Min-Max	21900 - 177063	26381 - 95129	
Mean	56637	57998	
Median (Q1,Q3)	50402 (38301, 69893)	59050 (38757, 69870)	
Quartile coefficient of dispersion	0.29	0.29	
**P02655 (Apolipoprotein C-II)**			0.676
Min-Max	285454 - 2779389	439282 - 2612294	
Mean	1233554	1358975	
Median (Q1,Q3)	1220672 (832580, 1545976)	1318040 (776211, 1915575)	
Quartile coefficient of dispersion	0.3	0.42	
**P07360 (Complement component C8 gamma chain)**			0.676
Min-Max	71604 - 194387	88949 - 176176	
Mean	128296	121937	
Median (Q1,Q3)	124165 (97681, 153105)	122447 (92327, 135582)	
Quartile coefficient of dispersion	0.22	0.19	
**P60709 (Actin, cytoplasmic 1)**			0.702
Min-Max	48263 - 599223	63952 - 167304	
Mean	127459	103533	
Median (Q1,Q3)	102339 (76794, 132788)	101029 (75637, 115115)	
Quartile coefficient of dispersion	0.27	0.21	
**P02750 (Leucine-rich alpha-2-glycoprotein)**			0.728
Min-Max	420416 - 2077668	513369 - 1524715	

Mean	921346	906435	
Median (Q1,Q3)	769216 (622024, 1036003)	895542 (674799, 1001048)	
Quartile coefficient of dispersion	0.25	0.19	
**P23142 (Fibulin-1)**			0.728
Min-Max	13662 - 146477	30077 - 77290	
Mean	54100	48261	
Median (Q1,Q3)	47121 (35606, 62920)	44779 (37215, 58466)	
Quartile coefficient of dispersion	0.28	0.22	
**Q9UGM5 (Fetuin-B)**			0.728
Min-Max	16848 - 179853	17713 - 42487	
Mean	32010	28690	
Median (Q1,Q3)	26212 (19320, 31940)	26365 (21536, 34213)	
Quartile coefficient of dispersion	0.25	0.23	
**P00739 (Haptoglobin-related protein)**			0.728
Min-Max	56516 - 342428	39598 - 210429	
Mean	142538	124050	
Median (Q1,Q3)	114205 (90902, 159989)	118435 (83866, 172409)	
Quartile coefficient of dispersion	0.28	0.35	
**P01700 (Immunoglobulin lambda variable 1-47)**			0.728
Min-Max	471410 - 1569918	552931 - 1834309	
Mean	930885	910702	
Median (Q1,Q3)	976471 (657377, 1107408)	741269 (668070, 974020)	
Quartile coefficient of dispersion	0.26	0.19	

**P00488 (Coagulation factor XIII A chain)**			0.754
Min-Max	40803 - 298684	37953 - 133492	
Mean	84991	83041	
Median (Q1,Q3)	72932 (58805, 90430)	75954 (61293, 101490)	
Quartile coefficient of dispersion	0.21	0.25	
**P02656 (Apolipoprotein C-III)**			0.754
Min-Max	956353 - 6179759	1202004 - 4729731	
Mean	3181629	2921688	
Median (Q1,Q3)	2994964 (2148835, 3891842)	2983209 (2016305, 3842254)	
Quartile coefficient of dispersion	0.29	0.31	
**P01591 (Immunoglobulin J chain)**			0.754
Min-Max	93924 - 555336	129424 - 448884	
Mean	291192	300076	
Median (Q1,Q3)	274798 (223424, 355471)	319557 (198203, 392958)	
Quartile coefficient of dispersion	0.23	0.33	
**P06276 (Cholinesterase)**			0.754
Min-Max	26394 - 1465373	51882 - 109425	
Mean	120524	78616	
Median (Q1,Q3)	72349 (60048, 84934)	75290 (58054, 102038)	
Quartile coefficient of dispersion	0.17	0.27	
**P80748 (Immunoglobulin lambda variable 3-21)**			0.754
Min-Max	44147 - 969193	137566 - 416147	

Mean	233640	226208	
Median (Q1,Q3)	191488 (146627, 256285)	190221 (150703, 280672)	
Quartile coefficient of dispersion	0.27	0.3	
**P49908 (Selenoprotein P)**			0.754
Min-Max	38237 - 436871	50346 - 122306	
Mean	97392	76655	
Median (Q1,Q3)	65358 (56500, 89581)	73533 (62816, 80624)	
Quartile coefficient of dispersion	0.23	0.12	
**P04264 (Keratin, type II cytoskeletal 1)**			0.781
Min-Max	76464 - 1002325	105372 - 685795	
Mean	261695	255455	
Median (Q1,Q3)	161526 (117155, 281222)	148322 (127482, 340751)	
Quartile coefficient of dispersion	0.41	0.46	
**P02765 (Alpha-2-HS-glycoprotein)**			0.781
Min-Max	578746 - 2121694	904277 - 2048533	
Mean	1305286	1382553	
Median (Q1,Q3)	1330048 (1049540, 1608700)	1356625 (1213288, 1474478)	
Quartile coefficient of dispersion	0.21	0.1	
**P02763 (Alpha-1-acid glycoprotein 1)**			0.781
Min-Max	1415079 - 14363021	3278150 - 7905878	
Mean	5463867	4921074	

Median (Q1,Q3)	5004828 (3304322, 7638226)	4797866 (3774699, 5475665)	
Quartile coefficient of dispersion	0.4	0.18	
**P00734 (Prothrombin)**			0.781
Min-Max	628951 - 1616387	706433 - 1225261	
Mean	911094	924178	
Median (Q1,Q3)	879807 (786299, 982053)	897439 (750928, 1050668)	
Quartile coefficient of dispersion	0.11	0.17	
**P27169 (Serum paraoxonase/arylesterase 1)**			0.781
Min-Max	122639 - 1358495	220147 - 796130	
Mean	597922	529421	
Median (Q1,Q3)	592470 (417405, 739776)	624099 (350295, 671850)	
Quartile coefficient of dispersion	0.28	0.31	
**P02753 (Retinol-binding protein 4)**			0.781
Min-Max	359920 - 1244208	525286 - 1107685	
Mean	793609	825009	
Median (Q1,Q3)	782139 (621801, 955093)	768382 (675525, 1017734)	
Quartile coefficient of dispersion	0.21	0.2	
**P55058 (Phospholipid transfer protein)**			0.781
Min-Max	5892 - 190083	11540 - 28787	
Mean	29716	19356	
Median (Q1,Q3)	19816 (15376, 25923)	19379 (15901, 21568)	

Quartile coefficient of dispersion	0.26	0.15	
**Q04756 (Hepatocyte growth factor activator)**			0.781
Min-Max	47498 - 230276	49760 - 127751	
Mean	98031	88052	
Median (Q1,Q3)	84197 (74655, 115162)	90637 (72531, 98433)	
Quartile coefficient of dispersion	0.21	0.15	
**P0DJI9 (Serum amyloid A-2 protein)**			0.781
Min-Max	10384 - 543615	25842 - 128217	
Mean	90602	58584	
Median (Q1,Q3)	62830 (29901, 83368)	53451 (34426, 66798)	
Quartile coefficient of dispersion	0.47	0.32	
**Q15848 (Adiponectin)**			0.808
Min-Max	19906 - 268709	29736 - 109999	
Mean	68666	57395	
Median (Q1,Q3)	53049 (43200, 73049)	55653 (38213, 65060)	
Quartile coefficient of dispersion	0.26	0.26	
**P22352 (Glutathione peroxidase 3)**			0.808
Min-Max	44875 - 472518	44001 - 350749	
Mean	187219	168882	
Median (Q1,Q3)	155151 (101448, 248373)	159041 (100751, 202804)	
Quartile coefficient of dispersion	0.42	0.34	
**P01857 (Immunoglobulin heavy constant gamma 1)**			0.835
Min-Max	7313244 - 18963559	7022801 - 16755579	

Mean	12504392	12490500	
Median (Q1,Q3)	12595150 (10380086, 14099032)	13172880 (10227547, 15389991)	
Quartile coefficient of dispersion	0.15	0.2	
**P01619 (Immunoglobulin kappa variable 3-20)**			0.835
Min-Max	806312 - 8712502	1777939 - 4792670	
Mean	2936996	2992028	
Median (Q1,Q3)	2961318 (2231497, 3480047)	2800204 (2229156, 3645730)	
Quartile coefficient of dispersion	0.22	0.24	
**P80108 (Phosphatidylinositol-glycan-specific phospholipase D)**			0.835
Min-Max	58961 - 555024	60888 - 213929	
Mean	120751	113906	
Median (Q1,Q3)	94988 (77940, 131195)	98592 (87693, 131476)	
Quartile coefficient of dispersion	0.25	0.2	
**A0A075B6K4 (Immunoglobulin lambda variable 3-10)**			0.835
Min-Max	8397 - 67450	8121 - 29645	
Mean	25046	20557	
Median (Q1,Q3)	19877 (15158, 27077)	21419 (14709, 27290)	
Quartile coefficient of dispersion	0.28	0.3	
**P01764 (Immunoglobulin heavy variable 3-23)**			0.835
Min-Max	17615 - 659115	28792 - 359774	
Mean	267600	228219	

Median (Q1,Q3)	233369 (86377, 377713)	252240 (164442, 318502)	
Quartile coefficient of dispersion	0.63	0.32	
**P04196 (Histidine-rich glycoprotein)**			0.862
Min-Max	491991 - 2013277	352235 - 1292273	
Mean	921604	863062	
Median (Q1,Q3)	814400 (725829, 1019125)	957812 (657050, 1009215)	
Quartile coefficient of dispersion	0.17	0.21	
**P06331 (Immunoglobulin heavy variable 4-34)**			0.862
Min-Max	6555 - 510978	16895 - 246278	
Mean	156453	136466	
Median (Q1,Q3)	131822 (88430, 211248)	138343 (94864, 178912)	
Quartile coefficient of dispersion	0.41	0.31	
**P01714 (Immunoglobulin lambda variable 3-19)**			0.862
Min-Max	12742 - 252877	53595 - 419243	
Mean	111073	141844	
Median (Q1,Q3)	108729 (79383, 126045)	107986 (92792, 131619)	
Quartile coefficient of dispersion	0.23	0.17	
**P07996 (Thrombospondin-1)**			0.889
Min-Max	30262 - 249894	79948 - 196782	
Mean	113320	112091	
Median (Q1,Q3)	100735 (63669, 160347)	94393 (85361, 126999)	
Quartile coefficient of dispersion	0.43	0.2	

**P04278 (Sex hormone-binding globulin)**			0.889
Min-Max	12801 - 66885	12983 - 54316	
Mean	37849	35606	
Median (Q1,Q3)	38686 (29614, 44860)	38755 (31487, 43729)	
Quartile coefficient of dispersion	0.2	0.16	
**A0A0A0MS15 (Immunoglobulin heavy variable 3-49)**			0.889
Min-Max	99354 - 611472	162607 - 303473	
Mean	289689	239816	
Median (Q1,Q3)	221906 (178489, 377616)	240134 (204156, 285233)	
Quartile coefficient of dispersion	0.36	0.17	
**P81605 (Dermcidin)**			0.889
Min-Max	5634 - 43358	9292 - 43612	
Mean	20913	20822	
Median (Q1,Q3)	17365 (10946, 29406)	15759 (12076, 25406)	
Quartile coefficient of dispersion	0.46	0.36	
**P04114 (Apolipoprotein B-100)**			0.917
Min-Max	486693 - 1819352	494705 - 3090469	
Mean	1046720	1219914	
Median (Q1,Q3)	1025301 (772255, 1299153)	979333 (946719, 1085279)	
Quartile coefficient of dispersion	0.25	0.07	
**P04040 (Catalase)**			0.917
Min-Max	30947 - 527917	34491 - 192571	

Mean	103807	94071	
Median (Q1,Q3)	76992 (63048, 117358)	90120 (64124, 108043)	
Quartile coefficient of dispersion	0.3	0.26	
**P12259 (Coagulation factor V)**			0.917
Min-Max	38963 - 1434382	87282 - 428787	
Mean	277851	225056	
Median (Q1,Q3)	213200 (126319, 340897)	212251 (153941, 255766)	
Quartile coefficient of dispersion	0.46	0.25	
**P30043 (Flavin reductase (NADPH))**			0.917
Min-Max	13615 - 151523	21985 - 70623	
Mean	47418	46106	
Median (Q1,Q3)	44956 (34686, 56619)	42820 (29335, 63955)	
Quartile coefficient of dispersion	0.24	0.37	
**P01023 (Alpha-2-macroglobulin)**			0.945
Min-Max	5663897 - 17389061	6091885 - 19034948	
Mean	10540923	11077769	
Median (Q1,Q3)	9573324 (8237088, 13281785)	9391288 (8861799, 11770539)	
Quartile coefficient of dispersion	0.23	0.14	
**O14791 (Apolipoprotein L1)**			0.945
Min-Max	145067 - 837572	153588 - 932194	
Mean	356526	382677	
Median (Q1,Q3)	313717 (249291, 435386)	327557 (248664, 405691)	

Quartile coefficient of dispersion	0.27	0.24	
**O75882 (Attractin)**			0.945
Min-Max	56336 - 294168	59593 - 281150	
Mean	111878	119459	
Median (Q1,Q3)	91775 (78925, 124044)	100489 (73227, 126127)	
Quartile coefficient of dispersion	0.22	0.27	
**P06702 (Protein S100-A9)**			0.945
Min-Max	4204 - 154075	7411 - 60053	
Mean	22510	22662	
Median (Q1,Q3)	15284 (12662, 23403)	14670 (12176, 29462)	
Quartile coefficient of dispersion	0.3	0.42	
**P61769 (Beta-2-microglobulin)**			0.972
Min-Max	12971 - 111447	17460 - 65665	
Mean	32892	33439	
Median (Q1,Q3)	28787 (22950, 38566)	29229 (24020, 36669)	
Quartile coefficient of dispersion	0.25	0.21	
**P03951 (Coagulation factor XI)**			0.972
Min-Max	29418 - 705129	31079 - 167666	
Mean	103260	72717	
Median (Q1,Q3)	52965 (44032, 90926)	51921 (45683, 88069)	
Quartile coefficient of dispersion	0.35	0.32	
**Q96KN2 (Beta-Ala-His dipeptidase)**			0.972
Min-Max	12607 - 76921	14798 - 44111	
Mean	35469	31653	

Median (Q1,Q3)	29801 (23123, 41642)	30656 (25411, 41937)	
Quartile coefficient of dispersion	0.29	0.25	
**P02747 (Complement C1q subcomponent subunit C)**			0.972
Min-Max	278887 - 1144412	421353 - 856772	
Mean	637509	627523	
Median (Q1,Q3)	628843 (502243, 748852)	640932 (524764, 711725)	
Quartile coefficient of dispersion	0.2	0.15	
**O43866 (CD5 antigen-like)**			0.972
Min-Max	3891 - 327793	5835 - 240375	
Mean	131415	127650	
Median (Q1,Q3)	137103 (45546, 197748)	114197 (73665, 193019)	
Quartile coefficient of dispersion	0.63	0.45	
**A0A0C4DH38 (Immunoglobulin heavy variable 5-51)**			0.999
Min-Max	26795 - 214408	36688 - 640544	
Mean	89096	142705	
Median (Q1,Q3)	74482 (58817, 113795)	82356 (65343, 93156)	
Quartile coefficient of dispersion	0.32	0.18	
**Q92954 (Proteoglycan 4)**			0.999
Min-Max	7366 - 84147	9463 - 54777	
Mean	29802	28486	
Median (Q1,Q3)	26892 (15145, 40513)	25885 (15427, 39307)	
Quartile coefficient of dispersion	0.46	0.44	

Q1 – the first quartile; Q3 – the third quartile; Mann-Whitney U test was applied for comparison of the quantitative data; p-value equal or less than 0.05 was considered statistically significant.
