# Supplementary material for: SWATH-MS for prospective identification of protein blood biomarkers of rtPA-associated intracranial hemorrhage in acute ischemic stroke: a pilot study
Source: Sci Rep. 2021 Sep 21;11:18765. doi: 10.1038/s41598-021-97710-9 (PMC8455557; doi:10.1038/s41598-021-97710-9)
Supplement: Supplementary file 4 — Supplementary Table 4. [file 41598_2021_97710_MOESM4_ESM.pdf]

SWATH- MS for prospective identification of protein blood biomarkers of rtPA-associated intracranial hemorrhage in acute ischemic stroke: a pilot study

Bartosz Karaszewski, Anna Gójska-Grymajło, Paulina Czaplewska, Bartosz Jabłoński, Aleksandra E. Lewandowska, Daria Ossowska, Adam Wyszomirski, Marek Hałas, Edyta Szurowska

Supplementary Table 2b. *Differences in distributions of* **plasma** *proteins in patients with and without intracranial hemorrhage (ICH).*

	Without ICH (N=32)	With ICH (N=9)	p value
**P04278 (Sex hormone-binding globulin)**			0.006
Min-Max	12364 - 77648	18890 - 137661	
Mean	32032	53615	
Median (Q1,Q3)	30979 (22554, 37261)	45947 (41476, 53836)	
Quartile coefficient of dispersion	0.25	0.13	
**P02675 (Fibrinogen beta chain)**			0.017
Min-Max	2599929 - 11512151	6803847 - 11978411	
Mean	7098058	9219731	
Median (Q1,Q3)	6691786 (5408873, 8878122)	9216882 (7608785, 10068706)	
Quartile coefficient of dispersion	0.24	0.14	
**P02679 (Fibrinogen gamma chain)**			0.021
Min-Max	4350465 - 15639932	9128520 - 14989155	
Mean	9465736	11716563	

Median (Q1,Q3)	9320935 (7574401, 11193242)	11465208 (10539057, 12945970)	
Quartile coefficient of dispersion	0.19	0.1	
**A0A0C4DH38 (Immunoglobulin heavy variable 5-51)**			0.021
Min-Max	26560 - 212136	52023 - 778336	
Mean	77448	169783	
Median (Q1,Q3)	58405 (49917, 78686)	103877 (85690, 121555)	
Quartile coefficient of dispersion	0.22	0.17	
**Q06033 (Inter-alpha-trypsin inhibitor heavy chain H3)**			0.025
Min-Max	86754 - 892388	208651 - 2060956	
Mean	245673	480053	
Median (Q1,Q3)	227784 (145420, 291568)	276340 (257465, 318183)	
Quartile coefficient of dispersion	0.33	0.11	
**P02649 (Apolipoprotein E)**			0.027
Min-Max	457437 - 1368153	431688 - 2074547	
Mean	819091	1233080	
Median (Q1,Q3)	784002 (679415, 981123)	1200818 (879202, 1547952)	
Quartile coefficient of dispersion	0.18	0.28	
**P36955 (Pigment epithelium-derived factor)**			0.038
Min-Max	139699 - 612807	311481 - 853268	
Mean	339511	441325	

Median (Q1,Q3)	321511 (262356, 403623)	408218 (366367, 444596)	
Quartile coefficient of dispersion	0.21	0.1	
**Q9NZP8 (Complement C1r subcomponent-like protein)**			0.055
Min-Max	26004 - 198813	33534 - 776229	
Mean	61450	125334	
Median (Q1,Q3)	56692 (46496, 65742)	44469 (39543, 52033)	
Quartile coefficient of dispersion	0.17	0.14	
**P81605 (Dermcidin)**			0.055
Min-Max	6708 - 90765	2224 - 92640	
Mean	22474	36337	
Median (Q1,Q3)	14264 (11281, 28442)	33314 (19872, 48967)	
Quartile coefficient of dispersion	0.43	0.42	
**P07357 (Complement component C8 alpha chain)**			0.063
Min-Max	107833 - 574584	176432 - 419414	
Mean	205794	248570	
Median (Q1,Q3)	194679 (169829, 222385)	220043 (203270, 239994)	
Quartile coefficient of dispersion	0.13	0.08	
**P02787 (Serotransferrin)**			0.068
Min-Max	8109647 - 23932972	9788805 - 17668437	
Mean	15794667	13285485	
Median (Q1,Q3)	15972430 (13265739, 18447993)	12904036 (10703028, 15435826)	

Quartile coefficient of dispersion	0.16	0.18	
**P00742 (Coagulation factor X)**			0.068
Min-Max	39427 - 141053	42625 - 224602	
Mean	72850	71128	
Median (Q1,Q3)	65385 (53521, 85184)	46212 (45875, 61769)	
Quartile coefficient of dispersion	0.23	0.15	
**Q15582 (Transforming growth factor-beta-induced protein ig-h3)**			0.073
Min-Max	6881 - 42951	16490 - 86108	
Mean	21358	33381	
Median (Q1,Q3)	19517 (13756, 28008)	28061 (17865, 34368)	
Quartile coefficient of dispersion	0.34	0.32	
**P02765 (Alpha-2-HS-glycoprotein)**			0.078
Min-Max	431299 - 1771046	1125631 - 1695680	
Mean	1088063	1346267	
Median (Q1,Q3)	1103596 (758902, 1356179)	1336773 (1127384, 1394154)	
Quartile coefficient of dispersion	0.28	0.11	
**P10643 (Complement component C7)**			0.083
Min-Max	71884 - 663613	175750 - 769549	
Mean	255777	358168	
Median (Q1,Q3)	242086 (189643, 312314)	307564 (251892, 400966)	
Quartile coefficient of dispersion	0.24	0.23	
**Q04756 (Hepatocyte growth factor activator)**			0.089

Min-Max	39962 - 384725	62400 - 501742	
Mean	84537	131031	
Median (Q1,Q3)	72939 (62773, 87375)	86483 (74361, 100947)	
Quartile coefficient of dispersion	0.16	0.15	
**P02751 (Fibronectin)**			0.101
Min-Max	110463 - 1991056	255294 - 1898594	
Mean	601258	911225	
Median (Q1,Q3)	505192 (313569, 793399)	776456 (625711, 1338264)	
Quartile coefficient of dispersion	0.43	0.36	
**P04196 (Histidine-rich glycoprotein)**			0.101
Min-Max	415218 - 1767001	497710 - 1961898	
Mean	783318	977359	
Median (Q1,Q3)	732850 (562310, 900453)	882510 (775691, 1033726)	
Quartile coefficient of dispersion	0.23	0.14	
**P01857 (Immunoglobulin heavy constant gamma 1)**			0.108
Min-Max	6398858 - 14836843	3663247 - 12344808	
Mean	10774948	9079869	
Median (Q1,Q3)	10851254 (9884772, 11934331)	10266224 (7916161, 11055019)	
Quartile coefficient of dispersion	0.09	0.17	
**P04040 (Catalase)**			0.108
Min-Max	46883 - 808154	47169 - 589779	
Mean	127072	130921	

Median (Q1,Q3)	88627 (71778, 113413)	63494 (57686, 105098)	
Quartile coefficient of dispersion	0.22	0.29	
**Q96PD5 (N-acetylmuramoyl-L-alanine amidase)**			0.115
Min-Max	39539 - 199252	97282 - 140890	
Mean	108000	122642	
Median (Q1,Q3)	112524 (96332, 126156)	128095 (115460, 129808)	
Quartile coefficient of dispersion	0.13	0.06	
**P60709 (Actin, cytoplasmic 1)**			0.123
Min-Max	50449 - 2140277	65909 - 308627	
Mean	154333	121433	
Median (Q1,Q3)	84909 (65096, 101896)	105752 (82293, 118050)	
Quartile coefficient of dispersion	0.22	0.18	
**P01700 (Immunoglobulin lambda variable 1-47)**			0.123
Min-Max	393248 - 1489365	436039 - 1161524	
Mean	814806	676260	
Median (Q1,Q3)	783016 (657674, 981802)	616186 (511789, 824750)	
Quartile coefficient of dispersion	0.2	0.23	
**Q13790 (Apolipoprotein F)**			0.123
Min-Max	13604 - 79353	16606 - 91246	
Mean	31685	41015	
Median (Q1,Q3)	27506 (21217, 35918)	38647 (28949, 45352)	
Quartile coefficient of dispersion	0.26	0.22	

**P00450 (Ceruloplasmin)**			0.131
Min-Max	694968 - 3572622	2053853 - 3599215	
Mean	2212000	2606122	
Median (Q1,Q3)	2278469 (1860124, 2623016)	2327312 (2251500, 2805438)	
Quartile coefficient of dispersion	0.17	0.11	
**P00738 (Haptoglobin)**			0.139
Min-Max	125094 - 35570728	4231652 - 13586900	
Mean	12989227	9551216	
Median (Q1,Q3)	12467390 (8856030, 16616488)	9425717 (8329927, 11898778)	
Quartile coefficient of dispersion	0.3	0.18	
**P04264 (Keratin, type II cytoskeletal 1)**			0.139
Min-Max	103159 - 773644	112738 - 715046	
Mean	283875	413853	
Median (Q1,Q3)	209338 (168292, 421144)	356162 (207466, 660444)	
Quartile coefficient of dispersion	0.43	0.52	
**P43251 (Biotinidase)**			0.139
Min-Max	29613 - 416652	37134 - 433061	
Mean	78765	95029	
Median (Q1,Q3)	66367 (56265, 79234)	57117 (46422, 58337)	
Quartile coefficient of dispersion	0.17	0.11	
**P07358 (Complement component C8 beta chain)**			0.147

Min-Max	42432 - 317497	107220 - 318705	
Mean	179169	226294	
Median (Q1,Q3)	173510 (131010, 213438)	217551 (163051, 301830)	
Quartile coefficient of dispersion	0.24	0.3	
**P35542 (Serum amyloid A-4 protein)**			0.147
Min-Max	170734 - 804320	187251 - 845550	
Mean	416364	528210	
Median (Q1,Q3)	408941 (297385, 496471)	514930 (399622, 638956)	
Quartile coefficient of dispersion	0.25	0.23	
**P05155 (Plasma protease C1 inhibitor)**			0.176
Min-Max	892556 - 4216511	2359601 - 4540704	
Mean	2632196	3102285	
Median (Q1,Q3)	2625369 (2268419, 3036883)	2856080 (2561660, 3366572)	
Quartile coefficient of dispersion	0.14	0.14	
**P01859 (Immunoglobulin heavy constant gamma 2)**			0.176
Min-Max	263387 - 3554534	1822449 - 3537976	
Mean	2200635	2731595	
Median (Q1,Q3)	2209864 (1579499, 3213859)	2814834 (2649962, 3054858)	
Quartile coefficient of dispersion	0.34	0.07	
**P18428 (Lipopolysaccharide-binding protein)**			0.197

Min-Max	47282 - 408017	121968 - 796290	
Mean	156426	241781	
Median (Q1,Q3)	149365 (116657, 180032)	163423 (134177, 204182)	
Quartile coefficient of dispersion	0.21	0.21	
**P06276 (Cholinesterase)**			0.197
Min-Max	31971 - 173555	47235 - 1470754	
Mean	76130	240499	
Median (Q1,Q3)	69436 (51367, 90692)	89122 (76543, 99636)	
Quartile coefficient of dispersion	0.28	0.13	
**P00740 (Coagulation factor IX)**			0.197
Min-Max	3734 - 138924	12536 - 61368	
Mean	23704	28281	
Median (Q1,Q3)	19812 (15019, 26463)	27790 (18477, 31454)	
Quartile coefficient of dispersion	0.28	0.26	
**P08603 (Complement factor H)**			0.208
Min-Max	426092 - 1505914	848817 - 2203213	
Mean	952226	1150990	
Median (Q1,Q3)	949090 (811351, 1055967)	1014026 (950785, 1052490)	
Quartile coefficient of dispersion	0.13	0.05	
**P00488 (Coagulation factor XIII A chain)**			0.208
Min-Max	42512 - 636897	49229 - 913390	
Mean	105417	185849	

Median (Q1,Q3)	90350 (66827, 106538)	105374 (86718, 111905)	
Quartile coefficient of dispersion	0.23	0.13	
**P04114 (Apolipoprotein B-100)**			0.219
Min-Max	367566 - 1536343	400100 - 2261530	
Mean	911969	1133218	
Median (Q1,Q3)	850452 (711516, 1128086)	1041452 (922611, 1299031)	
Quartile coefficient of dispersion	0.23	0.17	
**P10909 (Clusterin)**			0.219
Min-Max	728586 - 3189695	1550574 - 3386758	
Mean	2140037	2453760	
Median (Q1,Q3)	2102457 (1775252, 2458829)	2237078 (2158309, 2746152)	
Quartile coefficient of dispersion	0.16	0.12	
**P07225 (Vitamin K-dependent protein S)**			0.231
Min-Max	87605 - 405897	260051 - 755011	
Mean	286094	363667	
Median (Q1,Q3)	303492 (253964, 324241)	307150 (297831, 333180)	
Quartile coefficient of dispersion	0.12	0.06	
**P05452 (Tetranectin)**			0.231
Min-Max	38132 - 149225	49685 - 169895	
Mean	85149	100505	
Median (Q1,Q3)	83204 (60235, 103894)	94668 (78902, 111420)	

Quartile coefficient of dispersion	0.27	0.17	
**O14791 (Apolipoprotein L1)**			0.244
Min-Max	80053 - 469266	105058 - 734101	
Mean	242126	239885	
Median (Q1,Q3)	232958 (176803, 295702)	192362 (159354, 214474)	
Quartile coefficient of dispersion	0.25	0.15	
**P51884 (Lumican)**			0.244
Min-Max	117503 - 475138	182397 - 2199552	
Mean	259266	492931	
Median (Q1,Q3)	248512 (193288, 300842)	273409 (244597, 331222)	
Quartile coefficient of dispersion	0.22	0.15	
**P0C0L4 (Complement C4-A)**			0.257
Min-Max	59109 - 671783	195874 - 930713	
Mean	289040	388853	
Median (Q1,Q3)	302997 (189658, 357776)	316717 (270189, 392431)	
Quartile coefficient of dispersion	0.31	0.18	
**P01834 (Immunoglobulin kappa constant)**			0.257
Min-Max	5361350 - 28723564	9225106 - 24416188	
Mean	17723198	15276056	
Median (Q1,Q3)	16062868 (13035784, 23907197)	12659973 (11621238, 18735003)	
Quartile coefficient of dispersion	0.29	0.23	

**P08185 (Corticosteroid-binding globulin)**			0.257
Min-Max	111430 - 731478	259414 - 609671	
Mean	347686	401611	
Median (Q1,Q3)	351135 (283266, 406317)	342788 (324727, 481309)	
Quartile coefficient of dispersion	0.18	0.19	
**P27169 (Serum paraoxonase/arylesterase 1)**			0.270
Min-Max	102886 - 1542703	297575 - 1073647	
Mean	527220	610512	
Median (Q1,Q3)	476009 (308845, 696234)	611893 (490910, 633164)	
Quartile coefficient of dispersion	0.39	0.13	
**P01011 (Alpha-1-antichymotrypsin)**			0.284
Min-Max	295102 - 4856160	1983686 - 5006294	
Mean	2784498	3318850	
Median (Q1,Q3)	2751057 (2063687, 3695390)	2945480 (2810136, 3815903)	
Quartile coefficient of dispersion	0.28	0.15	
**P02652 (Apolipoprotein A-II)**			0.284
Min-Max	580239 - 4675204	1358009 - 4084919	
Mean	2342500	2774971	
Median (Q1,Q3)	2213564 (1807072, 3086422)	3001920 (1933869, 3632855)	
Quartile coefficient of dispersion	0.26	0.31	
**P05156 (Complement factor I)**			0.284

Min-Max	131774 - 614690	272249 - 638451	
Mean	326779	393357	
Median (Q1,Q3)	343309 (211347, 384158)	360788 (320394, 375960)	
Quartile coefficient of dispersion	0.29	0.08	
**P01782 (Immunoglobulin heavy variable 3-9)**			0.284
Min-Max	17656 - 145862	28264 - 745192	
Mean	53849	132024	
Median (Q1,Q3)	43502 (32338, 68124)	59358 (43892, 73521)	
Quartile coefficient of dispersion	0.36	0.25	
**P04003 (C4b-binding protein alpha chain)**			0.299
Min-Max	68853 - 1085186	368512 - 1664406	
Mean	656176	805768	
Median (Q1,Q3)	641605 (509450, 834899)	704772 (585577, 950119)	
Quartile coefficient of dispersion	0.24	0.24	
**O75882 (Attractin)**			0.299
Min-Max	25819 - 262834	62250 - 428148	
Mean	101069	144938	
Median (Q1,Q3)	90632 (65234, 106557)	95917 (82757, 114194)	
Quartile coefficient of dispersion	0.24	0.16	
**A0A075B6K4 (Immunoglobulin lambda variable 3-10)**			0.299
Min-Max	8875 - 113227	7469 - 41828	
Mean	24537	18865	

Median (Q1,Q3)	17873 (15250, 27177)	16468 (13321, 19688)	
Quartile coefficient of dispersion	0.28	0.19	
**P06681 (Complement C2)**			0.313
Min-Max	110305 - 590014	157878 - 447945	
Mean	211737	238124	
Median (Q1,Q3)	202764 (169684, 225590)	219595 (182008, 237081)	
Quartile coefficient of dispersion	0.14	0.13	
**P01721 (Immunoglobulin lambda variable 6-57)**			0.313
Min-Max	20640 - 155688	12900 - 501520	
Mean	46487	95685	
Median (Q1,Q3)	39070 (33626, 55364)	46939 (46600, 51193)	
Quartile coefficient of dispersion	0.24	0.05	
**P19652 (Alpha-1-acid glycoprotein 2)**			0.313
Min-Max	333676 - 2623202	612525 - 3064895	
Mean	1425755	1294236	
Median (Q1,Q3)	1400451 (1006661, 1789604)	1233688 (733502, 1269781)	
Quartile coefficient of dispersion	0.28	0.27	
**P15169 (Carboxypeptidase N catalytic chain)**			0.313
Min-Max	15720 - 345576	19622 - 204230	
Mean	45006	56504	
Median (Q1,Q3)	34567 (26952, 44773)	39744 (33673, 49627)	
Quartile coefficient of dispersion	0.25	0.19	

**P02748 (Complement component C9)**			0.345
Min-Max	259764 - 1144841	452607 - 1458914	
Mean	642724	758058	
Median (Q1,Q3)	614804 (546626, 733764)	634323 (614586, 782599)	
Quartile coefficient of dispersion	0.15	0.12	
**P02743 (Serum amyloid P-component)**			0.345
Min-Max	146313 - 803665	393077 - 1433988	
Mean	519268	652821	
Median (Q1,Q3)	496797 (374381, 667676)	519754 (476379, 662951)	
Quartile coefficient of dispersion	0.28	0.16	
**P06396 (Gelsolin)**			0.361
Min-Max	209206 - 720293	285290 - 750639	
Mean	480124	515742	
Median (Q1,Q3)	469028 (431968, 540223)	486715 (472188, 575988)	
Quartile coefficient of dispersion	0.11	0.1	
**P06702 (Protein S100-A9)**			0.361
Min-Max	3467 - 367227	6819 - 83340	
Mean	24309	23506	
Median (Q1,Q3)	10990 (7680, 14616)	15734 (8851, 19148)	
Quartile coefficient of dispersion	0.31	0.37	
**P02750 (Leucine-rich alpha-2-glycoprotein)**			0.378

Min-Max	280561 - 2399817	476000 - 1353170	
Mean	832665	862778	
Median (Q1,Q3)	752448 (581753, 848619)	754917 (741307, 1007670)	
Quartile coefficient of dispersion	0.19	0.15	
**P11226 (Mannose-binding protein C)**			0.378
Min-Max	13914 - 73974	12427 - 339713	
Mean	41307	65567	
Median (Q1,Q3)	36159 (28286, 53762)	28593 (25305, 44558)	
Quartile coefficient of dispersion	0.31	0.28	
**Q15848 (Adiponectin)**			0.378
Min-Max	18655 - 179730	33734 - 320244	
Mean	56824	86876	
Median (Q1,Q3)	52913 (34782, 72800)	54207 (38575, 87704)	
Quartile coefficient of dispersion	0.35	0.39	
**P35527 (Keratin, type I cytoskeletal 9)**			0.395
Min-Max	68545 - 501938	73286 - 646558	
Mean	161607	237918	
Median (Q1,Q3)	132886 (109020, 178337)	146160 (124126, 324161)	
Quartile coefficient of dispersion	0.24	0.45	
**P00747 (Plasminogen)**			0.395
Min-Max	344947 - 1380362	610850 - 1972420	
Mean	910589	931553	

Median (Q1,Q3)	881574 (760967, 1077137)	769266 (710835, 950520)	
Quartile coefficient of dispersion	0.17	0.14	
**P09871 (Complement C1s subcomponent)**			0.395
Min-Max	108067 - 371486	157218 - 402779	
Mean	218382	245088	
Median (Q1,Q3)	202683 (184702, 247982)	228654 (191716, 264005)	
Quartile coefficient of dispersion	0.15	0.16	
**P0DOY3 (Immunoglobulin lambda constant 3)**			0.395
Min-Max	18255 - 214262	70446 - 234211	
Mean	123944	143942	
Median (Q1,Q3)	121017 (93301, 160829)	153973 (88248, 161924)	
Quartile coefficient of dispersion	0.27	0.29	
**P49908 (Selenoprotein P)**			0.395
Min-Max	20288 - 451157	37768 - 1786365	
Mean	76908	256884	
Median (Q1,Q3)	63860 (47940, 85894)	70207 (63309, 79716)	
Quartile coefficient of dispersion	0.28	0.11	
**P00751 (Complement factor B)**			0.413
Min-Max	960740 - 2735945	1109437 - 2334895	
Mean	1477427	1547575	
Median (Q1,Q3)	1346865 (1189000, 1742790)	1567165 (1275634, 1690612)	

Quartile coefficient of dispersion	0.19	0.14	
**P02749 (Beta-2-glycoprotein 1)**			0.413
Min-Max	39240 - 1030631	377961 - 1562180	
Mean	588452	731262	
Median (Q1,Q3)	614986 (441308, 763410)	687315 (509321, 758810)	
Quartile coefficient of dispersion	0.27	0.2	
**P05090 (Apolipoprotein D)**			0.413
Min-Max	253786 - 1460193	367932 - 1869649	
Mean	587814	724853	
Median (Q1,Q3)	478390 (393323, 697281)	496090 (461017, 769361)	
Quartile coefficient of dispersion	0.28	0.25	
**P02655 (Apolipoprotein C-II)**			0.413
Min-Max	295522 - 3377463	512848 - 1662272	
Mean	1089278	1172480	
Median (Q1,Q3)	994302 (703092, 1392228)	1426395 (764990, 1558006)	
Quartile coefficient of dispersion	0.33	0.34	
**P02647 (Apolipoprotein A-I)**			0.431
Min-Max	13140959 - 46817478	16380844 - 43937132	
Mean	34825150	36129224	
Median (Q1,Q3)	35423221 (30670201, 39886506)	38442974 (37524322, 41000155)	
Quartile coefficient of dispersion	0.13	0.04	

**P01042 (Kininogen-1)**			0.431
Min-Max	567195 - 5732788	1653875 - 3097381	
Mean	2233417	2346402	
Median (Q1,Q3)	2050522 (1757456, 2584213)	2362545 (2039675, 2501430)	
Quartile coefficient of dispersion	0.19	0.1	
**P25311 (Zinc-alpha-2-glycoprotein)**			0.431
Min-Max	402477 - 1588930	613008 - 2153163	
Mean	1049927	1205383	
Median (Q1,Q3)	1029641 (767945, 1363843)	1093084 (940889, 1561449)	
Quartile coefficient of dispersion	0.28	0.25	
**Q9UK55 (Protein Z-dependent protease inhibitor)**			0.431
Min-Max	17533 - 136712	30586 - 240956	
Mean	57733	68930	
Median (Q1,Q3)	58693 (42215, 68405)	53598 (43177, 56303)	
Quartile coefficient of dispersion	0.24	0.13	
**P20742 (Pregnancy zone protein)**			0.431
Min-Max	5155 - 107858	11170 - 161144	
Mean	37640	42868	
Median (Q1,Q3)	25753 (17436, 48342)	14777 (12553, 63984)	
Quartile coefficient of dispersion	0.47	0.67	
**Q92954 (Proteoglycan 4)**			0.431
Min-Max	4370 - 91897	6997 - 48290	
Mean	20949	21781	

Median (Q1,Q3)	14599 (9132, 25642)	18007 (12541, 27053)	
Quartile coefficient of dispersion	0.47	0.37	
**P26927 (Hepatocyte growth factor-like protein)**			0.431
Min-Max	5388 - 74374	7402 - 106843	
Mean	19266	24996	
Median (Q1,Q3)	16770 (13330, 22478)	14131 (9998, 17317)	
Quartile coefficient of dispersion	0.26	0.27	
**Q14624 (Inter-alpha-trypsin inhibitor heavy chain H4)**			0.450
Min-Max	611294 - 3081254	1778669 - 2771681	
Mean	1980115	2242485	
Median (Q1,Q3)	2080659 (1598068, 2532355)	2311345 (2006396, 2424340)	
Quartile coefficient of dispersion	0.23	0.09	
**P02774 (Vitamin D-binding protein)**			0.469
Min-Max	671754 - 3301599	1378974 - 2302224	
Mean	1746611	1827069	
Median (Q1,Q3)	1710643 (1380582, 2013492)	1768512 (1612509, 1963019)	
Quartile coefficient of dispersion	0.19	0.1	
**P05546 (Heparin cofactor 2)**			0.469
Min-Max	290281 - 1446047	660936 - 2114124	
Mean	910930	1074553	
Median (Q1,Q3)	916967 (771885, 1064375)	955905 (781388, 1190278)	

Quartile coefficient of dispersion	0.16	0.21	
**P61769 (Beta-2-microglobulin)**			0.469
Min-Max	8824 - 131314	10037 - 209595	
Mean	29885	46791	
Median (Q1,Q3)	24131 (14692, 29930)	25809 (20311, 35316)	
Quartile coefficient of dispersion	0.34	0.27	
**P12259 (Coagulation factor V)**			0.469
Min-Max	36466 - 473193	62953 - 852378	
Mean	170149	237372	
Median (Q1,Q3)	135702 (63859, 235384)	147731 (92060, 267449)	
Quartile coefficient of dispersion	0.57	0.49	
**P01024 (Complement C3)**			0.488
Min-Max	1570059 - 10146209	4319991 - 7993289	
Mean	6201416	6703546	
Median (Q1,Q3)	6542065 (4959990, 7577238)	7099802 (6422460, 7409153)	
Quartile coefficient of dispersion	0.21	0.07	
**P04275 (von Willebrand factor)**			0.488
Min-Max	13112 - 214301	26462 - 111677	
Mean	63978	59148	
Median (Q1,Q3)	38692 (26946, 83714)	66266 (28966, 70902)	
Quartile coefficient of dispersion	0.51	0.42	
**P00915 (Carbonic anhydrase 1)**			0.488
Min-Max	40336 - 9384411	61566 - 656350	
Mean	423862	202986	

Median (Q1,Q3)	111386 (74301, 188890)	123910 (83770, 201078)	
Quartile coefficient of dispersion	0.44	0.41	
**P80748 (Immunoglobulin lambda variable 3-21)**			0.488
Min-Max	52309 - 461490	94764 - 2072862	
Mean	179045	386861	
Median (Q1,Q3)	160390 (123397, 233513)	174033 (138463, 240922)	
Quartile coefficient of dispersion	0.31	0.27	
**P02671 (Fibrinogen alpha chain)**			0.508
Min-Max	7478264 - 23758028	12731130 - 16956510	
Mean	14164009	14766440	
Median (Q1,Q3)	14402854 (11196350, 16065891)	14609937 (13244560, 15984800)	
Quartile coefficient of dispersion	0.18	0.09	
**P01019 (Angiotensinogen)**			0.508
Min-Max	111716 - 879236	557222 - 1189082	
Mean	591106	688067	
Median (Q1,Q3)	607907 (482768, 722793)	595735 (571566, 724296)	
Quartile coefficient of dispersion	0.2	0.12	
**P0C0L5 (Complement C4-B)**			0.508
Min-Max	26603 - 445014	89497 - 489096	
Mean	152350	181931	
Median (Q1,Q3)	144210 (75116, 182258)	145613 (129861, 169307)	

Quartile coefficient of dispersion	0.42	0.13	
**Q9UGM5 (Fetuin-B)**			0.508
Min-Max	11508 - 190623	14304 - 318028	
Mean	34773	56598	
Median (Q1,Q3)	28798 (21905, 36639)	25377 (17763, 31476)	
Quartile coefficient of dispersion	0.25	0.28	
**P09172 (Dopamine beta-hydroxylase)**			0.508
Min-Max	6208 - 60650	7227 - 44340	
Mean	25020	21208	
Median (Q1,Q3)	19945 (14199, 36018)	20077 (9037, 26591)	
Quartile coefficient of dispersion	0.43	0.49	
**P01023 (Alpha-2-macroglobulin)**			0.529
Min-Max	2791331 - 14238251	6909436 - 17564335	
Mean	9257932	10528031	
Median (Q1,Q3)	8613371 (7571907, 11472823)	10133962 (7043668, 12369203)	
Quartile coefficient of dispersion	0.2	0.27	
**P06727 (Apolipoprotein A-IV)**			0.529
Min-Max	747895 - 5768946	1194445 - 4638149	
Mean	2400659	2619502	
Median (Q1,Q3)	2289173 (1734716, 3067531)	2371660 (2273143, 2740083)	
Quartile coefficient of dispersion	0.28	0.09	
**P22792 (Carboxypeptidase N subunit 2)**			0.529
Min-Max	89005 - 651470	84480 - 306467	

Mean	180638	160919	
Median (Q1,Q3)	168535 (140249, 189897)	154972 (113824, 190186)	
Quartile coefficient of dispersion	0.15	0.25	
**P32119 (Peroxiredoxin-2)**			0.529
Min-Max	24284 - 7234159	23140 - 277332	
Mean	302822	106830	
Median (Q1,Q3)	71582 (46300, 93039)	94152 (47792, 120959)	
Quartile coefficient of dispersion	0.34	0.43	
**A0A0C4DH68 (Immunoglobulin kappa variable 2-24)**			0.529
Min-Max	38467 - 217485	65853 - 435244	
Mean	120203	157244	
Median (Q1,Q3)	115570 (94781, 162993)	123319 (105397, 158698)	
Quartile coefficient of dispersion	0.26	0.2	
**P01031 (Complement C5)**			0.550
Min-Max	96794 - 970896	499405 - 799509	
Mean	562814	604388	
Median (Q1,Q3)	547263 (490995, 627472)	545859 (529515, 702363)	
Quartile coefficient of dispersion	0.12	0.14	
**P19827 (Inter-alpha-trypsin inhibitor heavy chain H1)**			0.550
Min-Max	373145 - 3030206	945199 - 1832944	
Mean	1393496	1448581	

Median (Q1,Q3)	1327085 (1175751, 1686619)	1426166 (1344459, 1654618)	
Quartile coefficient of dispersion	0.18	0.1	
**P02538 (Keratin, type II cytoskeletal 6A)**			0.550
Min-Max	22121 - 81905	24105 - 93012	
Mean	49416	54188	
Median (Q1,Q3)	46365 (38797, 55521)	53838 (39278, 63237)	
Quartile coefficient of dispersion	0.18	0.23	
**P01602 (Immunoglobulin kappa variable 1-5)**			0.550
Min-Max	33486 - 642323	42540 - 184240	
Mean	99615	92719	
Median (Q1,Q3)	70729 (57449, 104792)	78988 (63834, 121928)	
Quartile coefficient of dispersion	0.29	0.31	
**Q08380 (Galectin-3-binding protein)**			0.550
Min-Max	22958 - 183469	41051 - 190648	
Mean	66573	81140	
Median (Q1,Q3)	59791 (46263, 74251)	61650 (48873, 97718)	
Quartile coefficient of dispersion	0.23	0.33	
**P43652 (Afamin)**			0.571
Min-Max	204006 - 960322	251946 - 946895	
Mean	515790	499623	
Median (Q1,Q3)	491020 (389399, 659816)	444267 (405853, 543166)	
Quartile coefficient of dispersion	0.26	0.14	

**P02746 (Complement C1q subcomponent subunit B)**			0.571
Min-Max	62098 - 509991	87684 - 545470	
Mean	146211	181113	
Median (Q1,Q3)	134641 (119979, 155148)	140234 (128702, 158955)	
Quartile coefficient of dispersion	0.13	0.11	
**P05543 (Thyroxine-binding globulin)**			0.592
Min-Max	76984 - 783157	140189 - 631261	
Mean	222319	254468	
Median (Q1,Q3)	202397 (165394, 259498)	234328 (170783, 238721)	
Quartile coefficient of dispersion	0.22	0.17	
**P80108 (Phosphatidylinositol-glycan-specific phospholipase D)**			0.592
Min-Max	41905 - 516452	49029 - 183678	
Mean	97019	96184	
Median (Q1,Q3)	79105 (65464, 104517)	87312 (72399, 100328)	
Quartile coefficient of dispersion	0.23	0.16	
**P17936 (Insulin-like growth factor-binding protein 3)**			0.592
Min-Max	10671 - 83451	16659 - 146877	
Mean	36509	42380	
Median (Q1,Q3)	33833 (26562, 42627)	32069 (25109, 35857)	
Quartile coefficient of dispersion	0.23	0.18	
**P01009 (Alpha-1-antitrypsin)**			0.614
Min-Max	9957658 - 25154761	7212953 - 19853089	

Mean	15294376	14916802	
Median (Q1,Q3)	15625758 (13624533, 16610395)	17440604 (13033110, 18702564)	
Quartile coefficient of dispersion	0.1	0.18	
**P02790 (Hemopexin)**			0.614
Min-Max	2343793 - 9418916	4972845 - 7536397	
Mean	6302251	6223038	
Median (Q1,Q3)	6443158 (5490465, 7611252)	6406561 (5606483, 6623049)	
Quartile coefficient of dispersion	0.16	0.08	
**P01871 (Immunoglobulin heavy constant mu)**			0.614
Min-Max	258835 - 9420666	1200838 - 5985130	
Mean	3249709	2799336	
Median (Q1,Q3)	2785544 (1963089, 4083297)	2762409 (1520173, 2902667)	
Quartile coefficient of dispersion	0.35	0.31	
**P02763 (Alpha-1-acid glycoprotein 1)**			0.614
Min-Max	873115 - 10413871	1227035 - 6872573	
Mean	3994428	3369421	
Median (Q1,Q3)	3529565 (2365995, 4858256)	2995582 (1722094, 4445104)	
Quartile coefficient of dispersion	0.34	0.44	
**P08697 (Alpha-2-antiplasmin)**			0.614
Min-Max	154784 - 575277	218334 - 584634	
Mean	308665	297531	

Median (Q1,Q3)	268811 (238587, 358924)	251206 (228044, 310870)	
Quartile coefficient of dispersion	0.2	0.15	
**P35858 (Insulin-like growth factor-binding protein complex acid labile subunit)**			0.614
Min-Max	66821 - 209513	71899 - 355364	
Mean	126487	136586	
Median (Q1,Q3)	121246 (92501, 155275)	109360 (87534, 152331)	
Quartile coefficient of dispersion	0.25	0.27	
**P03952 (Plasma kallikrein)**			0.614
Min-Max	103880 - 268151	90497 - 298158	
Mean	182442	189562	
Median (Q1,Q3)	178628 (149702, 205771)	191292 (176586, 197334)	
Quartile coefficient of dispersion	0.16	0.06	
**P02745 (Complement C1q subcomponent subunit A)**			0.614
Min-Max	211715 - 851096	233638 - 586558	
Mean	389558	347277	
Median (Q1,Q3)	331632 (293002, 445707)	337162 (273894, 383708)	
Quartile coefficient of dispersion	0.21	0.17	
**O43866 (CD5 antigen-like)**			0.614
Min-Max	3480 - 322991	5495 - 410872	
Mean	84633	75018	
Median (Q1,Q3)	43668 (7282, 116921)	28363 (7093, 60649)	

Quartile coefficient of dispersion	0.88	0.79	
**P01008 (Antithrombin-III)**			0.637
Min-Max	686696 - 3053989	1117568 - 1888834	
Mean	1442060	1455312	
Median (Q1,Q3)	1410175 (1138089, 1684062)	1510970 (1273287, 1582768)	
Quartile coefficient of dispersion	0.19	0.11	
**P04217 (Alpha-1B-glycoprotein)**			0.637
Min-Max	784767 - 2830160	1377984 - 2080077	
Mean	1816931	1759325	
Median (Q1,Q3)	1800820 (1574774, 2088878)	1758820 (1669808, 1804369)	
Quartile coefficient of dispersion	0.14	0.04	
**P68871 (Hemoglobin subunit beta)**			0.637
Min-Max	686651 - 56397097	1045548 - 12069212	
Mean	6696125	5855620	
Median (Q1,Q3)	3863538 (2335323, 6791359)	4098570 (3002776, 9545698)	
Quartile coefficient of dispersion	0.49	0.52	
**P13645 (Keratin, type I cytoskeletal 10)**			0.637
Min-Max	72281 - 453467	36222 - 483570	
Mean	227626	217941	
Median (Q1,Q3)	216553 (144119, 299036)	173810 (105877, 297077)	
Quartile coefficient of dispersion	0.35	0.47	

**P00736 (Complement C1r subcomponent)**			0.637
Min-Max	138169 - 384868	168126 - 975321	
Mean	220273	304762	
Median (Q1,Q3)	213758 (181675, 250726)	225545 (174579, 273733)	
Quartile coefficient of dispersion	0.16	0.22	
**A0A0B4J1V0 (Immunoglobulin heavy variable 3-15)**			0.637
Min-Max	188962 - 3580527	521827 - 1332424	
Mean	798757	792180	
Median (Q1,Q3)	724544 (582151, 845985)	747556 (584082, 861816)	
Quartile coefficient of dispersion	0.18	0.19	
**P02533 (Keratin, type I cytoskeletal 14)**			0.637
Min-Max	31409 - 1652624	53725 - 1381918	
Mean	144150	234337	
Median (Q1,Q3)	89070 (65419, 107513)	66809 (55853, 106020)	
Quartile coefficient of dispersion	0.24	0.31	
**P01619 (Immunoglobulin kappa variable 3-20)**			0.659
Min-Max	736309 - 7451024	1197981 - 4024721	
Mean	2701506	2457316	
Median (Q1,Q3)	2636005 (1888727, 3178117)	2280211 (2076552, 2792819)	
Quartile coefficient of dispersion	0.25	0.15	

**O75636 (Ficolin-3)**			0.659
Min-Max	75125 - 250861	117927 - 421417	
Mean	153101	185548	
Median (Q1,Q3)	149641 (134478, 175297)	143727 (131957, 196763)	
Quartile coefficient of dispersion	0.13	0.2	
**P02768 (Serum albumin)**			0.682
Min-Max	28032024 - 99664127	22380121 - 65615663	
Mean	47354612	43743827	
Median (Q1,Q3)	46028851 (37443012, 54244733)	47775165 (31713569, 48737833)	
Quartile coefficient of dispersion	0.18	0.21	
**P02741 (C-reactive protein)**			0.682
Min-Max	22432 - 566541	23875 - 338987	
Mean	88469	110931	
Median (Q1,Q3)	47497 (36702, 98885)	34703 (32617, 151804)	
Quartile coefficient of dispersion	0.46	0.65	
**P06312 (Immunoglobulin kappa variable 4-1)**			0.682
Min-Max	265532 - 935607	407135 - 1017303	
Mean	574686	562211	
Median (Q1,Q3)	577032 (433523, 703772)	507600 (423351, 639438)	
Quartile coefficient of dispersion	0.24	0.2	
**P01034 (Cystatin-C)**			0.682
Min-Max	9482 - 161425	10622 - 155054	

Mean	23426	33032	
Median (Q1,Q3)	16393 (13320, 23376)	17719 (16030, 20574)	
Quartile coefficient of dispersion	0.27	0.12	
**P29622 (Kallistatin)**			0.705
Min-Max	84241 - 465974	157068 - 718604	
Mean	203151	254951	
Median (Q1,Q3)	193432 (166386, 247791)	204199 (181730, 228364)	
Quartile coefficient of dispersion	0.2	0.11	
**P13671 (Complement component C6)**			0.705
Min-Max	121851 - 515388	125052 - 1035644	
Mean	246018	307689	
Median (Q1,Q3)	239602 (193557, 286836)	234046 (183774, 281701)	
Quartile coefficient of dispersion	0.19	0.21	
**P0DJI8 (Serum amyloid A-1 protein)**			0.705
Min-Max	24595 - 855040	42378 - 418314	
Mean	218919	177286	
Median (Q1,Q3)	185535 (95199, 321921)	167735 (129886, 187397)	
Quartile coefficient of dispersion	0.54	0.18	
**P02775 (Platelet basic protein)**			0.705
Min-Max	16008 - 481447	40780 - 178811	
Mean	119083	92657	
Median (Q1,Q3)	82132 (38038, 140891)	80290 (55512, 115986)	

Quartile coefficient of dispersion	0.57	0.35	
**P03951 (Coagulation factor XI)**			0.705
Min-Max	27132 - 1468952	18315 - 142094	
Mean	100128	66269	
Median (Q1,Q3)	44327 (39717, 62279)	54846 (40059, 86309)	
Quartile coefficient of dispersion	0.22	0.37	
**O00391 (Sulfhydryl oxidase 1)**			0.729
Min-Max	11373 - 66481	13184 - 49262	
Mean	24598	24505	
Median (Q1,Q3)	20296 (16255, 26934)	20942 (18738, 27001)	
Quartile coefficient of dispersion	0.25	0.18	
**P00739 (Haptoglobin-related protein)**			0.753
Min-Max	27990 - 303332	58781 - 362243	
Mean	121338	140527	
Median (Q1,Q3)	111784 (83297, 135255)	121875 (85099, 159402)	
Quartile coefficient of dispersion	0.24	0.3	
**P02042 (Hemoglobin subunit delta)**			0.753
Min-Max	23278 - 10436111	23761 - 398739	
Mean	448230	137749	
Median (Q1,Q3)	76628 (34310, 161382)	84618 (53065, 222472)	
Quartile coefficient of dispersion	0.65	0.61	
**P30043 (Flavin reductase (NADPH))**			0.753
Min-Max	10270 - 1273221	17374 - 219201	
Mean	82113	59590	

Median (Q1,Q3)	38050 (27725, 56089)	34326 (25482, 63507)	
Quartile coefficient of dispersion	0.34	0.43	
**Q9Y5Y7 (Lymphatic vessel endothelial hyaluronic acid receptor 1)**			0.753
Min-Max	8735 - 28054	6794 - 35107	
Mean	16219	16542	
Median (Q1,Q3)	14791 (10919, 18995)	13350 (9755, 22276)	
Quartile coefficient of dispersion	0.27	0.39	
**P00734 (Prothrombin)**			0.777
Min-Max	396342 - 1660623	668348 - 1835673	
Mean	928970	988007	
Median (Q1,Q3)	903629 (752977, 1022297)	949764 (740362, 1073406)	
Quartile coefficient of dispersion	0.15	0.18	
**P02760 (Protein AMBP)**			0.777
Min-Max	61778 - 626727	84436 - 727914	
Mean	284815	314674	
Median (Q1,Q3)	223780 (119321, 454357)	290735 (203702, 369897)	
Quartile coefficient of dispersion	0.58	0.29	
**P05154 (Plasma serine protease inhibitor)**			0.777
Min-Max	62853 - 274752	74586 - 200879	
Mean	115325	117695	
Median (Q1,Q3)	106489 (86753, 130261)	110113 (96068, 124228)	
Quartile coefficient of dispersion	0.2	0.13	

**P22352 (Glutathione peroxidase 3)**			0.777
Min-Max	49394 - 447170	76987 - 352490	
Mean	127780	133073	
Median (Q1,Q3)	114402 (90594, 134393)	104954 (89583, 120105)	
Quartile coefficient of dispersion	0.19	0.15	
**P00746 (Complement factor D)**			0.777
Min-Max	4302 - 56386	8413 - 72536	
Mean	31209	34952	
Median (Q1,Q3)	31612 (19900, 41575)	29909 (27319, 38850)	
Quartile coefficient of dispersion	0.35	0.17	
**P01714 (Immunoglobulin lambda variable 3-19)**			0.777
Min-Max	18228 - 253280	14727 - 500977	
Mean	97443	124783	
Median (Q1,Q3)	91907 (55001, 128059)	86643 (59645, 107553)	
Quartile coefficient of dispersion	0.4	0.29	
**B9A064 (Immunoglobulin lambda-like polypeptide 5)**			0.801
Min-Max	38557 - 166014	54533 - 162337	
Mean	95351	95870	
Median (Q1,Q3)	90160 (65563, 124358)	80105 (71353, 130290)	
Quartile coefficient of dispersion	0.31	0.29	
**P69905 (Hemoglobin subunit alpha)**			0.801
Min-Max	868136 - 50482776	1214276 - 12813464	
Mean	6562716	5650107	

Median (Q1,Q3)	4193688 (2774389, 7550733)	4271130 (4074870, 7579690)	
Quartile coefficient of dispersion	0.46	0.3	
**P02753 (Retinol-binding protein 4)**			0.801
Min-Max	176654 - 968292	370054 - 1553806	
Mean	595266	627154	
Median (Q1,Q3)	582634 (399298, 777399)	527065 (418721, 643313)	
Quartile coefficient of dispersion	0.32	0.21	
**P04433 (Immunoglobulin kappa variable 3-11)**			0.801
Min-Max	39811 - 1749424	31505 - 1309506	
Mean	506378	544457	
Median (Q1,Q3)	351603 (291033, 648879)	478676 (176585, 749585)	
Quartile coefficient of dispersion	0.38	0.62	
**P01591 (Immunoglobulin J chain)**			0.825
Min-Max	70304 - 475081	129300 - 737689	
Mean	252858	305383	
Median (Q1,Q3)	222495 (187591, 336359)	309285 (167870, 336492)	
Quartile coefficient of dispersion	0.28	0.33	
**P04004 (Vitronectin)**			0.850
Min-Max	732255 - 4014260	1989228 - 4547688	
Mean	2532227	2672483	

Median (Q1,Q3)	2519122 (2165203, 3116609)	2373868 (2074791, 3090188)	
Quartile coefficient of dispersion	0.18	0.2	
**P07996 (Thrombospondin-1)**			0.850
Min-Max	31761 - 226887	42492 - 687359	
Mean	81288	128844	
Median (Q1,Q3)	60815 (45783, 103738)	58771 (51860, 70020)	
Quartile coefficient of dispersion	0.39	0.15	
**P35908 (Keratin, type II cytoskeletal 2 epidermal)**			0.850
Min-Max	26993 - 246505	29124 - 219308	
Mean	80894	89136	
Median (Q1,Q3)	66049 (50214, 93056)	61613 (45458, 128155)	
Quartile coefficient of dispersion	0.3	0.48	
**P23142 (Fibulin-1)**			0.850
Min-Max	17059 - 260486	25357 - 84356	
Mean	57490	53191	
Median (Q1,Q3)	49300 (38185, 62412)	51782 (35651, 62148)	
Quartile coefficient of dispersion	0.24	0.27	
**Q96IY4 (Carboxypeptidase B2)**			0.850
Min-Max	30638 - 218775	37628 - 194563	
Mean	56912	65979	
Median (Q1,Q3)	53132 (43192, 57187)	48791 (45112, 60427)	
Quartile coefficient of dispersion	0.14	0.15	
**P00918 (Carbonic anhydrase 2)**			0.850

Min-Max	32832 - 1153001	37516 - 404354	
Mean	104343	97682	
Median (Q1,Q3)	61155 (49758, 75301)	59446 (43741, 84608)	
Quartile coefficient of dispersion	0.2	0.32	
**P07360 (Complement component C8 gamma chain)**			0.850
Min-Max	63446 - 186566	82044 - 441089	
Mean	112665	145205	
Median (Q1,Q3)	105030 (94693, 135506)	106724 (90203, 136534)	
Quartile coefficient of dispersion	0.18	0.2	
**P01860 (Immunoglobulin heavy constant gamma 3)**			0.850
Min-Max	30469 - 2668165	108534 - 1409029	
Mean	615205	486433	
Median (Q1,Q3)	436266 (138056, 872351)	394641 (208774, 615142)	
Quartile coefficient of dispersion	0.73	0.49	
**A0A0A0MS15 (Immunoglobulin heavy variable 3-49)**			0.850
Min-Max	61855 - 537830	173413 - 491203	
Mean	252660	253586	
Median (Q1,Q3)	224874 (171901, 299817)	233345 (176919, 268137)	
Quartile coefficient of dispersion	0.27	0.2	
**P01764 (Immunoglobulin heavy variable 3-23)**			0.850
Min-Max	13140 - 515754	34153 - 609350	

Mean	171251	174754	
Median (Q1,Q3)	171353 (38379, 221712)	119000 (43533, 162329)	
Quartile coefficient of dispersion	0.7	0.58	
**P19823 (Inter-alpha-trypsin inhibitor heavy chain H2)**			0.875
Min-Max	561680 - 3140585	1127813 - 1974590	
Mean	1619183	1619636	
Median (Q1,Q3)	1633633 (1383034, 1818187)	1717461 (1369483, 1920851)	
Quartile coefficient of dispersion	0.14	0.17	
**P02656 (Apolipoprotein C-III)**			0.875
Min-Max	618186 - 6155578	1113354 - 4012052	
Mean	2756885	2765953	
Median (Q1,Q3)	2601740 (2064648, 3567330)	3298675 (2246997, 3544985)	
Quartile coefficient of dispersion	0.27	0.22	
**Q16610 (Extracellular matrix protein 1)**			0.875
Min-Max	30717 - 244097	39114 - 348549	
Mean	75194	98958	
Median (Q1,Q3)	68012 (51023, 84138)	68293 (50103, 94285)	
Quartile coefficient of dispersion	0.25	0.31	
**P00748 (Coagulation factor XII)**			0.875
Min-Max	60604 - 328916	122857 - 497249	
Mean	172230	194083	

Median (Q1,Q3)	170256 (143669, 191070)	153374 (134980, 192411)	
Quartile coefficient of dispersion	0.14	0.18	
**A0A0B4J2H0 (Immunoglobulin heavy variable 1-69D)**			0.875
Min-Max	33030 - 474154	52030 - 563916	
Mean	86732	124937	
Median (Q1,Q3)	67393 (58251, 93247)	77225 (60282, 86316)	
Quartile coefficient of dispersion	0.23	0.18	
**P55058 (Phospholipid transfer protein)**			0.875
Min-Max	7232 - 122028	6817 - 115840	
Mean	20572	25279	
Median (Q1,Q3)	14096 (12038, 20300)	14304 (11731, 18624)	
Quartile coefficient of dispersion	0.26	0.23	
**P02654 (Apolipoprotein C-I)**			0.900
Min-Max	394398 - 2166309	340976 - 2683947	
Mean	1121837	1190162	
Median (Q1,Q3)	1099236 (699419, 1507592)	1125341 (859666, 1244324)	
Quartile coefficient of dispersion	0.37	0.18	
**Q96KN2 (Beta-Ala-His dipeptidase)**			0.900
Min-Max	8248 - 96818	12320 - 62291	
Mean	25614	25544	
Median (Q1,Q3)	21070 (14301, 29279)	21446 (17433, 28526)	
Quartile coefficient of dispersion	0.34	0.24	

**P02766 (Transthyretin)**			0.925
Min-Max	318673 - 4267647	647967 - 2539277	
Mean	1539814	1419254	
Median (Q1,Q3)	1272113 (753616, 1986297)	1298911 (1005514, 1601701)	
Quartile coefficient of dispersion	0.45	0.23	
**P06331 (Immunoglobulin heavy variable 4-34)**			0.925
Min-Max	11013 - 280243	10690 - 262154	
Mean	84533	90893	
Median (Q1,Q3)	68550 (38998, 103029)	77092 (36759, 98524)	
Quartile coefficient of dispersion	0.45	0.46	
**P0DJI9 (Serum amyloid A-2 protein)**			0.925
Min-Max	11198 - 563242	19577 - 77743	
Mean	73145	40478	
Median (Q1,Q3)	31374 (22458, 83807)	37347 (24049, 48593)	
Quartile coefficient of dispersion	0.58	0.34	
**P07477 (Trypsin-1)**			0.925
Min-Max	9378 - 273432	21554 - 138544	
Mean	57277	44014	
Median (Q1,Q3)	31782 (25020, 53000)	30264 (25528, 44023)	
Quartile coefficient of dispersion	0.36	0.27	
**P01861 (Immunoglobulin heavy constant gamma 4)**			0.950
Min-Max	13235 - 681793	13492 - 182712	
Mean	156994	103201	

Median (Q1,Q3)	100923 (46842, 167279)	106589 (63385, 137862)	
Quartile coefficient of dispersion	0.56	0.37	
**P13796 (Plastin-2)**			0.950
Min-Max	18236 - 252191	48977 - 155515	
Mean	99399	95269	
Median (Q1,Q3)	104039 (31122, 132905)	102750 (60855, 114914)	
Quartile coefficient of dispersion	0.62	0.31	
**P02747 (Complement C1q subcomponent subunit C)**			0.950
Min-Max	140280 - 828841	351194 - 746599	
Mean	551755	555043	
Median (Q1,Q3)	541258 (506186, 629067)	583980 (510514, 598619)	
Quartile coefficient of dispersion	0.11	0.08	
**P01876 (Immunoglobulin heavy constant alpha 1)**			0.999
Min-Max	37173 - 17707384	5316074 - 14032357	
Mean	7726159	8141267	
Median (Q1,Q3)	7352056 (6125429, 9641325)	6951089 (5668007, 9718803)	
Quartile coefficient of dispersion	0.22	0.26	
**A0A0C4DH72 (Immunoglobulin kappa variable 1-6)**			0.999
Min-Max	288827 - 2537372	451928 - 1141652	
Mean	749277	703085	
Median (Q1,Q3)	659071 (537379, 818344)	684106 (632299, 777604)	

Quartile coefficient of dispersion	0.21	0.1	

Q1 – the first quartile; Q3 – the third quartile; Mann-Whitney U test was applied for comparison of the quantitative data; p-value equal or less than 0.05 was considered statistically significant.
